# When Lone Wolf Defectors Undermine the Power of the Opt-Out Default

**DOI:** 10.1038/s41598-020-65163-1

**Published:** 2020-06-02

**Authors:** Eamonn Ferguson, Ruslan Shichman, Jonathan H. W. Tan

**Affiliations:** 1 School of Psychology, University of Nottingham, Nottingham, NG7 2RD UK; 2 Centre for Decision Research and Experimental Economics (CeDEx), University of Nottingham, Nottingham, NG7 2RD UK; 3 Department of Economics, School of Social Sciences, Nanyang Technological University, 48 Nanyang Avenue, Singapore, 639818 Singapore

**Keywords:** Evolution, Psychology

## Abstract

High levels of cooperation are a central feature of human society, and conditional cooperation has been proposed as one proximal mechanism to support this. The counterforce of free-riding can, however, undermine cooperation and as such a number of external mechanisms have been proposed to ameliorate the effects of free-riding. One such mechanism is setting cooperation as the default (i.e., an opt-out default). We posit, however, that in dynamic settings where people can observe and condition their actions on others’ behaviour, ‘lone wolf’ defectors undermine initial cooperation encouraged by an opt-out default, while ‘good shepherds’ defeat the free-riding encouraged by an opt-in default. Thus, we examine the dynamic emergence of conditional cooperation under different default settings. Specifically, we develop a game theoretical model to analyse cooperation under defaults for cooperation (opt-out) and defection (opt-in). The model predicts that the ‘lone wolf’ effect is stronger than the ‘good shepherd’ effect, which – if anticipated by players – should strategically deter free-riding under opt-out and cooperation under opt-in. Our experimental games confirm the existence of both ‘lone wolf’ defectors and ‘good shepherd’ cooperators, and that the ‘lone wolf’effect is stronger in the context of organ donation registration behaviour. We thus show a potential ‘dark side’ to conditional cooperation (‘lone wolf effect’) and draw implications for the adoption of an opt-out organ donation policy.

## Introduction

The high levels of cooperation observed across all human societies^1–4^ are hard to explain from the perspective of natural selection: why would someone perform a behaviour that benefits another at a personal cost^3,4^? To address this problem a number of ultimate (‘why’) explanations (e.g., kin selection), supported by a number of proximal (‘how’) mechanisms, have been proposed^2–7^. This paper focuses on one of these proximal mechanisms: conditional cooperation^6^. Conditional cooperators are individuals who vary their levels of cooperation (e.g., contributing to the public good) proportionally to the contributions of others^6,8^. However, while the number of conditional cooperators is high (62%), their contribution is proportionally less than one-to-one with the group average and this combined with more free-riders (18.7%) than rare unconditional cooperators results in average contributions decaying over time^8–10^. As such, other mechanisms are required to ameliorate this decay and sustain cooperation^1^. For example, setting defaults supporting wider societal cooperation^11^ which have been successful for organ donation^5^, childhood and adult vaccinations^12,13^, and environmental conservation^14^ is one option. To date, the majority of work on the power of defaults has examined this in static games where choices are made simultaneously^15,16^. Rarely has it been studied in dynamic games^17^ which model real world settings where others’ choices can be observed and strategically responded to. We contribute to this literature by examining how the cooperative context (opt-in or opt-out defaults for organ donation) shapes the dynamic expression of conditional cooperation. We show that initial cooperation, encouraged by an opt-out default, is *undermined* by the free-rider (‘lone wolf’) who leads with unilateral defection (we call this the ‘lone wolf’ effect). We show how initial free-riding, supported by an opt-in default, is *defeated* by the unconditional cooperator (‘good shepherd’) who leads with unilateral cooperation (we call this the ‘good shepherd’ effect). We also show that the ‘lone wolf’ effect is stronger than the ‘good shepherd’ effect.

## Cooperative defaults and the dynamics of conditional cooperation

When faced with alternative choices, the default is the option selected by the person if they do not *actively* take action^18^. Within the context of cooperation, the use of opt-out defaults has been a powerful mechanism to increase cooperation in contexts characterized by free-riding^5,11–14,19^, for example in organ donation, an application which motivated the games studied in this paper^5,19^. Under an opt-out policy for organ donation, the *default* is that everyone is presumed to be a donor unless they *actively* opt-out. Conversely, the opt-in policy for organ donation supports free-riding, with the *default* for everyone to be a non-donor unless they *actively* opt-in and register with about 38% of the UK and 60% of the USA population registering under opt-in. However, moving to an opt-out, from an opt-in, policy increases cooperation in terms of the number of registered donors and the number of potential organs for transplantation^5,19,20^.

Defaults are effective policy tools because choosing the default: (1) requires *reduced effort* (e.g. reduced cognitive load in the face of complexity), (2) biases the decision maker’s behaviour towards the *status quo* response, and (3) frames the perceived meaning of the behaviour^11,18,21^. Applied to organ donation, the opt-out default makes registration *easy* with everyone registered and thus preserves the status quo. Furthermore, the meaning of organ donation under opt-out is that of a mundane and everyday act of cooperation (equivalent to letting someone ahead of you in a queue)^21^. Similarly for opt-in, remaining unregistered is the *easy* option with the status quo being that the majority remain unregistered, which leads to a context supporting free-riding. Furthermore, registering to donate an organ (cooperating) is perceived as an *extreme cooperative* act (similar to giving away 50% on one’s wealth to charity after death) under opt-in^21^ with the opt-in context characterised by reduced trust that others will cooperate^22^. We provide evidence from a survey experiment conducted over two waves (Supplementary File S1) confirming the differential *status quo* perceptions for opt-out and opt-in, with participants perceiving more people as remaining registered under opt-out (73.1% _wave 1_ & 72.6% _wave 2_) compared to registering under opt-in (29.8% _wave 1_ & 32.8% _wave 2_). People are also more trusting that others will remain registered under opt-out than register under opt-in. Thus, the opt-out default is seen as having a large number of people trusted to remain as donor, and the opt-in default as one with a few people trusted to register.

Given the very different meanings of the opt-out and opt-in defaults, how will this influence the emergence of conditional cooperation to  affect overall levels of cooperation and free-riding? The effect of the default on the emergence of conditional cooperation will critically depend on whether or not the behaviour is observable^6,7^.

### Observability and feedback

Cooperation is more likely to emerge if people can observe others cooperate and respond contingently^7^. With limited information about others’ cooperative choices we would expect the influence of conditional cooperation to be small and for an opt-out default to encourage cooperation and the opt-in default to encourage defection^23^. For example, in the context of organ donation, health authorities provide *generic* information only in terms of the number of donors or organs needed and thus people are not able to respond contingently to others’ registration decisions. Consistent with this lack of opportunity to respond contingently, an *opt-out advantage* of organ donation has been observed in terms of increased numbers of transplants^5,19,20^. However, if people can observe others’ decisions to register or de-register immediately, they can change their behaviour contingent on others actions. With such *individualistic feedback*, we would expect to observe strong effects of conditional cooperation. Indeed, the use of social media, allowing people to share their organ donor status and positive or negative stories about organ donation in real time means that the potential for such individualistic feedback is a reality these days^24–29^. A recent *Facebook* campaign exemplifies effects of individualistic feedback on organ donor registrations^25^. Set within an opt-in system this campaign allowed individuals to post status updates about their organ donor registration, resulting in increased registrations^25^. This so called ‘Facebook’ effect indicates how with an opt-in system and individualistic feedback, conditional cooperation can result in increased cooperation (our ‘good shepherd’ effect). We develop this by experimentally testing the effects of individualistic feedback. In particular, our experiment contrasts *static games* where feedback is provided only across games (no individualistic feedback) to *dynamic games* where subjects can also respond contingently to the feedback of others’ actions within a game before payoffs accrue. We further explore how such feedback varies as a function of default setting (opt-in vs opt-out).

### Opt-out vs Opt-in and the role of lone wolves and good shepherds

What happens to levels of cooperation or defection when people *can observe others’ decisions*, when these are framed within either cooperation (opt-out) or free-riding (opt-in) defaults?

Being registered to donate organs under either an opt-in or opt-out system involves *psychological costs*^30,31^. These costs stem from considering one’s *morality*, perceived violations of *bodily integrity*, *medical mistrust* at how one may be treated by the medical profession in life-threatening situations (e.g., harvesting organs before death), the *‘ick’ factor* (i.e., donating organs feels disgusting), the *‘jinx’ factor* (i.e., registering makes one more likely to die) and a preference for *individual* over *state ownership*. However, while it is individually costly to be a registered donor, the person also benefits more with increased numbers of others registered, as more organs will be available in times of need. Maintaining the status quo of an opt-out default yields a Pareto efficient state with a higher organ supply, while the status quo of an opt-in default, with a lower organ supply, is socially inefficient. As people opt-out (opt-in), the supply of organ and related benefits decrease (increase), resulting in Pareto inferior (superior) states compared to the status quo. Thus, organ donation and public goods games have similar payoff structures ex ante^32^.

The psychological cost of organ donation implies that it pays off (and is therefore incentive compatible) to deregister, under an opt-out system, when others are observed to deregister. Consistently, the behavioural rule of conditional cooperation also predicts that people will deregister when others do (negative conditional cooperation). We term this the ‘lone wolf effect’ as others deregister contingent on the actions of a deregistering ‘lone wolf’. Conditional cooperation similarly predicts that people will register if others do (positive conditional cooperation)^6^. As discussed above, donating one’s organs is perceived as an extreme act of cooperation under an opt-in system^21^ and thus will signal the person’s virtue as a ‘good shepherd’ that others could follow and likewise cooperate and register^33^. The ‘good shepherd’ effect is compatible with the positive ‘Facebook’ effect discussed above. However, it pays off to *not* follow when others are observed to register to avoid incurring psychological costs. Thus, the ‘lone wolf’ will have a stronger negative impact on cooperation than the positive impact of a ‘good shepherd’. Also, there are more free-riders than unconditional cooperators to trigger off the ‘lone wolf’ effect^8,34^.

These decisions can be analysed with a simple game as follows, which forms the basis of our study. A full game theoretic analysis and proofs are found in Supplementary File S2. Consider players who have to choose whether or not to change their registration status from being unregistered under opt-in or registered under an opt-out. Registration decreases payoffs, as it incurs psychological costs. One’s expected payoff increases when co-players register, as it increases the chance of transplant and survival after organ failure. In static games, defaults do not change the dominant strategy of free riding. In dynamic games with an opt-in policy, *not* following a ‘good shepherd’ who has opted in dominates the strategy of following to opt-in, because being registered is psychologically costly. There is no incentive to be a ‘good shepherd’ and the equilibrium is to stay (mutually) unregistered. Conversely, in dynamic games with an opt-out policy, following a ‘lone wolf’ who had opted-out dominates the psychologically costly strategy of staying registered. This ultimately results in mutual deregistering, which decreases expected payoffs. However, anticipating a worse eventual outcome from opting out, by backward induction one is better off sticking to the default (in equilibrium). Moreover, one need not worry about being free ridden on if defection by others can be observed and responded to before payoffs accrue; one can therefore wait to see if that really happens instead of defecting on the belief that others will defect too. This establishes the *strategic* power of the default embodied in opt-out, which is absent in static games. However, if others deviate off equilibrium, in particular free-riders opt-out and unconditional cooperators in opt-in, it pays to follow a ‘lone-wolf’ but *not* a ‘good shepherd’. We show that contrary to equilibrium predictions under self-interest, the ‘good shepherd’ increases cooperation by leading conditional cooperators thereby defeating free riding, and cooperative behaviour as signalled by an opt-out default policy is undermined over time when conditional cooperators can observe and follow ‘lone-wolf’ defection.

### Cooperative and defection spill-over effects

Cooperative defaults can be changed in practice. For organ donation most countries have started to move to an opt-out policy having previously had an opt-in policy^19^. What is the effect of this change? Evidence shows that exposure to a cooperative context enhances subsequent generosity (a ‘cooperative spill-over’ effect)^35^. Conversely, exposure to a non-cooperation context leads to a ‘defection’ mind-set and increases subsequent free-riding (a ‘defection spill-over’ effect)^36^. We examine such spill-over effects by exploring how the experience of a change in default (from opt-in to opt-out and visa-versa) influences the dynamics of cooperation. The opt-in default should lead to a defection mind-set that spills over and, via a strong ‘lone-wolf’ effect, reduces the potential benefits of the opt-out policy. Therefore, we hypothesise that a move from opt-in to opt-out will result in reduced registration rates in the presence of individualised feedback.

## Method

### Sample

Two hundred and thirteen participants took part in the economic game experiment (*M* age = 21.76; *SD* = 2.84; 62% female; 27% on the organ donor register and 83% believed that the game represented organ donor registrations). They were recruited via the University of Nottingham CeDEx subject pool.

### Procedure and design

**Figure 1:**
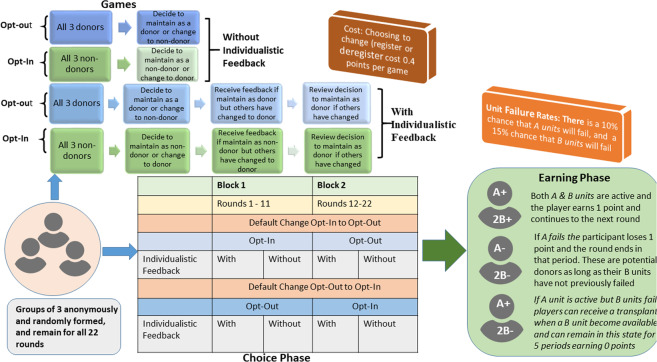
Schematic of the experimental games and design features.

Participants played one game in each *round*. There were 22 rounds in each experimental session. In each round, participants made registration choices. We use the term *period* to refer to each time point within a round. A schematic of the procedure and design is presented in Fig. 1, which shows that all registration choices were made in the *choice phase* which precede the *earnings phase* where individuals then realize their health outcomes on organ failure, transplantations or death.

Our experiment extends the basic static opt-in game of Kessler and Roth^32^, see also^37^ (see Supplementary File S3 for full verbatim instructions and screen shots of the game). As with the original studies^32^, we use induced value to model incentives relevant to organ donation. Extending the study by  Kessler and Roth^32^ we test an opt-out static game, as well as introduce dynamic opt-in and opt-out games. These dynamic games differ by providing individualistic feedback that allows players to update and change their choices contingent on others’ actions in multi-period choice tasks within a game. This models conditional cooperation in organ donation choices in reality. Our experiment also differs in that we explore dynamic changes in default setting (participants play a block of 11 opt-in games and move to play a block of 11 opt-out or vice versa). Finally, we let participants find out their *health outcomes* (i.e., whether or not they actually donate or need and receive an organ), ergo *experimental earnings*, only at the end of the experiment. Thus, we temporally ensure that all registration decisions precede health outcomes as would happen in reality. In Kessler and Roth’s experiment^32^, participants can choose to register or not and then they find out if they need organs or have died and can donate before making their next registration decision. Thus, those who have experienced life or death as a donor or recipient in a previous round make living decisions anew. Their learning patterns and behaviour are, therefore, potentially influenced by their experiences of health outcomes as donor or recipient, which do not feature in reality. To avoid this, we have participants make and receive per-round feedback on registration choices for all their *choice tasks*, after which they find out their health outcomes in corresponding *earnings stages* from four rounds randomly selected by the computer to determine their payments (see Fig. 1).

Participants made registration choices in 22 rounds of *choice tasks* in groups of three with fixed and anonymous membership. Before making choices, participants were told that they would start each round with one active *A unit* two active *B units*, which models a good state of health with functioning brain and kidneys, respectively, and an *initial endowment* of 2 points. Each point was worth 0.75p (UK). The task was to choose their *donor status*, which was whether or not to register or remain as a donor. Being registered or remaining registered as a donor cost 0.4 points.

A 2 (*default change*: opt-in to opt-out or opt-out to opt-in) by 2 (*order of default change*: opt-in to opt-out vs opt-out to opt-in) by 2 (*feedback*: with or without individualistic feedback) mixed design was used. *Default change* was a within-subject factor and *order of default change* and *individualistic feedback* a between-subject factors. Twelve lab-based sessions were organized into 6 sessions (3 with individualistic feedback and 3 without) starting with opt-out (11 rounds in block 1) and changing to opt-in (11 rounds block 2) and 6 sessions (3 with individualistic feedback and 3 without) starting with opt-in (11 rounds in block 1) and changing to opt-out (11 rounds in block 2). Participants were not informed how many rounds to expect but were instructed on the new rules of the game when moving from one default to another. Randomization was at the session level and within session for with and without feedback. The experiment was programmed in *z*-Tree^38^. Participants were incentive compatibly paid according to the protocol described.

While our game’s incentives are similar to voluntary contribution mechanism (VCM) experiments, in that individually costly contributions improve expected social welfare, there are three main differences in that our game: (1) has non-deterministic payoffs that depend on probabilistic events in one’s simulated life-cycle in the earnings stage; (2) has feedback within games, which implies a novel dynamic move structure, and (3) is framed within specific defaults. However, we would still expect to see some key features of (1) static VCM experiments where defaults and frames influence cooperation^15,16^, (2) repeated VCM experiments by observing a decay in contributions over rounds^9,10^, and (3) dynamic VCM experiments where feedback within games yield conditional cooperation or conflict^17,39^.

### Manipulation of defaults frame

Under *opt-in* participants were told that ‘At the start of each round, everyone in the group is automatically a non-donor.’ In contrast, under the *opt-out* participants were told that ‘At the start of each round, everyone in the group is automatically a donor.’ Thus, we frame defaults in a similar way to others in this field of research^21,37^.

### Manipulation of feedback

Following Tan *et al*.^17^ *individualistic feedback* on others’ cooperation was provided to allow others to conditionally respond. The screen showed the current number of other members of their group who were registered as donors. This initially stated that all three were donors under opt-out, and all three were non-donors under opt-in. This was updated per-period as and when someone in the group had changed their status. Based on that individualistic feedback, participants could change their status from the default as a donor under opt-out and non-donor under-opt-in. We prevented strategic cheap talk or non-committal actions by disallowing participants to revert after changing status (i.e., each player could change their status once), thus allowing for direct interpretation of the data without confounds from such motives, and to ensure timely progress in the experiment. This design feature was also motivated by the evidence that people very rarely change their organ donor registrations decisions. For example, 6 months before Wales in the UK moved to opt-out, 1% of those registered under opt-in stated they would deregister under opt-out^40^.

Participants could change their status as long as someone had changed their status in the previous period within that round. The choice task for each round lasts at least one period (if no player changes or all players change in the first period) and at most three periods (if one player changes in each period). As there were three players in each group one could change their status in the first period if two others had changed in period 1, in the second period if one other had changed in the preceding period, or in the third period if one other had changed in each of the two preceding periods. Only final decisions factored into the earnings stage.

In games *without individualistic feedback*, participants only knew others’ choices at the end of each round when choices were fixed and payoffs had accrued. Therefore, this required only one period and did not allow for within round conditional cooperation.

### Lifecycle earnings

The earnings phase followed after completion of all 22 rounds of choice tasks in the choice phase (see Fig. 1). In each earnings stage, a participant started with one active *A unit* (akin to a brain or heart), two active *B units* (akin to kidneys). Each earnings stage proceeded in periods, and registration decisions corresponded with the choices made in the respective rounds of the choice phase. In each period, there is a 10% chance that their *A unit* would fail, and a 15% chance that their *B units* would fail (both *B units fail* at the same time). If their *A unit* and *B units* are active in a period, they earn 1 point and the round continues to the next period. If their *A unit* fails, they lose 1 point and the round ends in that period. If their *A unit* is still active but *B units* fail, they can continue for up to 5 periods without *active B units* while waiting for a donor *B unit*. Before receiving a donor B unit, a participant will earn 0 points per period. They can receive a *donor B* unit from a co-participant who is a donor whose *A unit* has failed but *B units* have not failed before. If in one of these periods a participant receives a *B unit* from someone else, he or she will start earning points again. If one does not receive a *B unit* within those five periods, he/she loses 1 point and their round ends in the last of those five periods. On average participants earned £12.77 (= US$16.73) for around an hour’s worth of work.

### Outcome

Our main outcome variable was individual registration choice where 1 = registered, 0 = not registered, i.e. those actively opted in or those not actively opted out. Participants made decisions by pressing a button to *maintain* with the default (registered under opt-out or non-registered under opt-in) or one to *change* away from the default (de-register under opt-out and register under opt-in). Thus, they were making active choices about whether to be a donor or not.

### Covariates

For robustness, we assessed a number of covariates that may influence organ donor registration decisions. We asked participants at the end of the game to indicate ‘yes’ or ‘no’ to whether they were currently registered as an organ donor and to indicate if they felt that the game represented (1) organ donation, (2) blood donation, (3) donating money to charity, (4) giving up time to volunteer for a charity (coded 1 = organ donation and 0 = others). We asked about current and past blood donor status, and other volunteering behaviour. To avoid over-fitting our models we only held being on the organ donor register as a covariate as it is highly correlated with blood donor and volunteering status^41^. Participants completed the attitudes toward organ donation questionnaire^30^. This was not analysed due to low reliability of some of the sub-scales (e.g., α = 0.45 & 0.55).

### Ethics statement

All three experiments were approved and registered by the ethical procedures of the School of Psychology Ethic committee at the University of Nottingham (Experiments 1 reference 543, Experiment 2 reference 550, Experiment 3 reference 410) and were conducted in accordance with the relevant guidelines and regulations of the British Psychological Society (https://www.bps.org.uk/sites/bps.org.uk/files/Policy/Policy%20-%20Files/BPS%20Code%20of%20Ethics%20and%20Conduct%20%28Updated%20July%202018%29.pdf) and the University of Nottingham (https://workspace.nottingham.ac.uk/display/ResEth/Code+of+Research+Conduct+and+Research+Ethics?preview = /123507321/298524330/Code%20of%20Research%20Conduct%20and%20Research%20Ethics%20(Version%206a)%20(revisions%20Mar%202019).docx). Participants in all three experiments providing signed informed consent with their anonymity assured. Experiment 3 was pre-registered (OSF: see https://osf.io/g6mct/).

## Results

**Figure 2:**
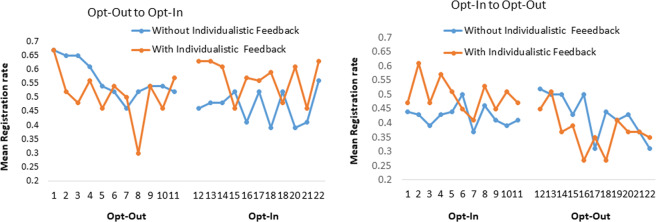
Effects of Policy Change as a Function of Feedback for the 22 Rounds.

**Figure 3:**
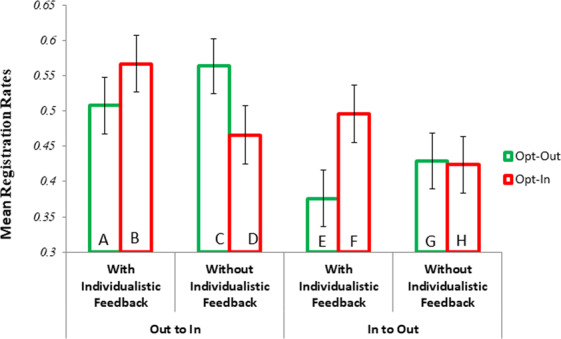
Mean Registration Rate by Condition. Bars A to D refer to the conditions when participants moved from opt-out to opt-in and the bars E to H for participants who moved from an opt-in to opt-out. The results are further split by feedback type. Means and Standard Errors (se) for this figure can be found in Supplementary File S4 Table S1. Table S2 in Supplementary File S4 details regression models designed to assess spill-over effects in this table. Error bars are 95% CIs.

Figure 2 details the dynamic time trends for registration decisions under changes from opt-in and opt-out and visa-versa, with or without individualistic feedback and Fig. 3 shows these as means (see Supplementary File S4 Table S1 for more details). Descriptively, it can be seen that the move from opt-in to opt-out results in a steep decline in registration rates, especially with individualistic feedback. Conversely, moving from opt-out to opt-in with individualistic feedback results in an initial uplift in registration rates. This may reflect a psychological contrast effect whereby people have become accustomed to observing others defecting by deregistering under opt-out, but suddenly observing others cooperate may be morally elevating and inspire cooperation^33^. This effect is worthy of future exploration.

### Spill-over effects

Before examining our main hypotheses regarding conditional cooperation and the ‘lone wolf’ and ‘good shepherd’ effects, there is evidence for a number of *spill-over effects* that are noteworthy. As predicted moving from opt-in to opt-out, with individualistic feedback, results in overall lower registrations under opt-out (Bar E in Fig. 2) than in opt-in (Bar F in Fig. 2). Regression analysis confirms this decrease in registration rates from opt-in to opt-out with individualistic feedback is significant (Supplementary File S4 Table S2: *B* = −0.124, *SE* = 0.062, *p* = 0.043, *95%CI* = −0.245,–0.004 without covariates and *B* = −0.131, *SE* = 0.067, *p* = 0.050, *95%CI* = −0.263, −0.000 with covariates). We explore below the ‘lone wolf’ effect as an explanation for this decrease. Also, the move from opt-out to opt-in without individualistic feedback results in a significant reduction in registration rates (moving from Bar C to Bar D: *B* = −0.103, *SE* = 0.045; *p* = 0.024, *95%CI* = −0.192, −0.014: without covariates and *B* = −0.178, *SE* = 0.061; *p* = 0.004, *95%CI* = −0.298, −0.058 with covariates). This reflects the reverse of the standard opt-out advantage and may be referred to as an *opt-in disadvantage* that is observed specifically when individualistic feedback is not possible.

### Main analytical strategy

To account for the non-independence of repeated group interactions, we analyse group means as the main dependent variable, using Generalized Least Squares (GLS) with group level clustered random errors. All models include *Round* (round 1–22) and default (*Opt-In*: 1 = opt-in 0 = opt-out), and control for within group age, sex, belief that the game is about organ donation (Believe it is a game about organ donation = 1, all other beliefs =0) and if currently registered as a donor (1 = register, 0 = not). The main results for conditional cooperation reported below hold when the same analyses are conducted without covariates^42^ (Tables S3, S4 and S5 in Supplementary File S4) and with individual decisions instead of group level means as the unit of analysis both with and without covariates (see Table S6 to S11 in Supplementary File S4).

### Replicating the ‘opt-out advantage’ and ‘facebook’ effects

Model 1 (Table 1) explores the overall effect of the presence of individualistic feedback and its interaction with default (Feedback * Opt-In). The significant negative effect for round shows a decay in cooperation over time, consistent with VCM experiments. Furthermore, the negative effect of the *Opt-In* replicates general finding that overall registration rates are higher under opt-out compared to opt-in, potentially leading to more transplantations, i.e. the overall *‘Opt-Out Advantage*’^5,19,20^. The positive and significant interaction of feedback by default (Feedback * Opt-In) indicates that the effect of individualistic feedback is stronger under opt-in than opt-out overall, replicating the ‘Facebook’ effect^25^. This could result in more cooperation (registrations) in opt-in than opt-out under individualistic feedback overall, which shall be corroborated by Model 5.

**Table 1:** Overall (Model 1) and Conditional Cooperation (Model 2) Effects (Random Effects GLS Models with group level clustered random error) on group averages of decisions to register plus Covariates. The dependent variable is the group average of the Decision to Register (for each individual, 1 = on the register: actively registered under opt-in and not opted-out under opt-out, or 0 = not on the register: not having registered under opt-in and opted-out under opt-out). We have the following independent variables. Round is the game number 1–22. Feedback: 0 = without individualistic feedback and 1 = with individualistic feedback, Opt-In: 0 = Opt-out and = 1 = Opt-in. Lag Round Decisions = percent of group registered in the previous round. Sex: Female = 0, Male = 1. Game Beliefs: Not believe it is a game about organ donation = 0, Believe it is a game about organ donation = 1, Organ Donor Register: 0 if report not currently registered and 1 if currently registered.

Decision to Register	Coefficient (SE_robust_)	p	95%		Coefficient (SE_robust_)	p	95%	
			Lower	Upper			Lower	Upper
	Model 1				Model 2			
Round	**−0.004 (0.002)**	**0.043**	**−0.008**	**−0.000**	−0.002 (0.001)	0.203	−0.004	0.001
Feedback	−0.072 (0.064)	0.256	−0.197	0.053	−0.043 (0.038)	0.252	−0.117	0.031
Opt-In	**−0.051 (0.023)**	**0.023**	**−0.096**	**−0.007**	−0.020 (0.013)	0.137	−0.046	0.006
Lag Round Decisions					**0.427 (0.060)**	**0.000**	**0.310**	**0.544**
Feedback* Opt-In	**0.141 (0.039)**	**0.000**	**0.065**	**0.217**	**0.083 (0.026)**	**0.001**	**0.032**	**0.135**
Age	0.000(0.025)	0.984	−0.048	0.049	0.005 (0.015)	0.732	−0.024	0.034
Sex	0.142 (0.127)	0.262	−0.107	0.391	0.113 (0.077)	0.141	−0.038	0.265
Game Beliefs	0.103 (0.139)	0.462	−0.171	0.376	0.053 (0.090)	0.551	−0.123	0.229
Organ Donor Register	0.035(0.126)	0.782	−0.212	0.282	0.020 (0.088)	0.824	−0.153	0.192
Constant	0.396 (0.542)	0.465	−0.667	1.458	0.098 (0.341)	0.774	−0.571	0.767
R^2^_overall_	0.033				0.214			
N of Observations (Groups)	3,102 (71)				2,961 (71)			

### Conditional cooperation (‘lone wolf’ effect) across rounds

To model the effects of conditional cooperation across rounds Model 2 (Table 1) adds the lagged effect of other’s decision in the previous round (*Lag Round Decisions*). Consistent with previous research on conditional cooperation we observe a positive effect^6,8–10^ showing that overall people respond conditionally to cooperation overall decisions in the previous round by increasing their cooperation.

### Lone-wolf effect across rounds

Models 3 and 4 in Table 2 further explore the effect of across round conditional cooperation by examining it as a function of default (Lag Round Decisions * Opt-In) either without individualistic feedback (Model 3) or with individualistic feedback (Model 4). The positive and significant conditional cooperation effect of decisions in previous rounds is found in both models. In Model 3 (without individualistic feedback) the significant and negative interaction effect indicates that when individualistic feedback is not present, (negative) conditional cooperation is stronger under opt-out. That is, without individualistic feedback, observing previous round cooperation leads to more conditional cooperation in the present round under opt-out. Model 4 shows no difference between opt-in and opt-out in terms of across round conditional cooperation when individualistic feedback is present, which is possibly diminished by the more prominent role that within round conditional cooperation plays, as we test next.

**Table 2:** Across Round Conditional Cooperation Effects as a function of Default and Feedback (Random Effects GLS Models with group level clustered random error) on group averages of decisions to register plus Covariates - the across round Lone-Wolf Effect. The dependent variable is the group average of the Decision to Register (for each individual, 1 = on the register: actively registered under opt-in and not opted-out under opt-out, or 0 = not on the register: not having registered under opt-in and opted-out under opt-out). We have the following independent variables. Round is the game number 1–22, Opt-In: 0 = Opt-out and = 1 = Opt-in. Lag Round Decisions = percent of group registered in the previous round. Sex: Female = 0, Male = 1. Game Beliefs: Not believe it is a game about organ donation = 0, Believe it is a game about organ donation = 1, Organ Donor Register: 0 if report not currently registered and 1 if currently registered.

Decision to Register	Without Individualistic Feedback				With Individualistic Feedback			
	Coefficient (SE_robust_)	p	95%		Coefficient (SE_robust_)	p	95%	
			Lower	Upper			Lower	Upper
	Model 3				Model 4			
Round	−0.001 (0.001)	0.346	−0.003	0.001	−0.002 (0.002)	0.296	−0.006	0.002
Opt-In	**0.052 (0.024)**	**0.030**	**0.005**	**0.100**	0.014 (0.040)	0.716	−0.063	0.092
Lag Round Decisions	**0.695 (0.060)**	**0.000**	**0.577**	**0.813**	**0.318 (0.087)**	**0.000**	**0.147**	**0.488**
Lag Round Decisions* Opt-In	**−0.129 (0.050)**	**0.010**	**−0.228**	**−0.031**	0.111 (0.086)	0.196	−0.057	0.278
Age	0.000 (0.015)	0.991	−0.029	0.028	0.012 (0.022)	0.585	−0.030	0.054
Sex	−0.001 (0.087)	0.989	−0.171	0.169	0.152 (0.105)	0.148	−0.054	0.358
Game Beliefs	0.036 (0.067)	0.595	−0.096	0.167	0.045 (0.131)	0.732	−0.211	0.301
Organ Donor Register	0.048 (0.060)	0.422	−0.069	0.165	0.001 (0.133)	0.995	−0.260	0.262
Constant	0.115 (0.327)	0.724	−0.525	0.755	−0.041 (0.472)	0.931	−0.967	0.885
R^2^_overall_	0.403				0.186			
N of Observations(Groups)	756 (36)				2,205 (35)			

### Lone-wolf effect within round

We hypothesized that a stronger ‘lone-wolf’ effect would be observed when people could update their behaviour based on feedback of others’ behaviour before each round ends and payoffs accrue. Model 5 (Table 3) tests this by examining how observations of decisions in previous periods within rounds (*Lag Period Decisions*) influences present period decisions and how this is moderated by the default setting (Lag Period Decisions * Opt-In). The results show that *Opt-In* is positive and significant corroborating that under an opt-in setting individualistic feedback results in more cooperation relative to opt-out^25^. The positive coefficient for *Lag Period Decisions* provides evidence of conditional cooperation within rounds. Furthermore, the significant interaction term Lag Period Decisions * Opt-In is negative indicating relatively less conditional cooperation within rounds under opt-in compared to opt-out. That is, consistent with the hypothesized ‘lone-wolf’ effect people are more likely to conditionally cooperate and deregister under opt-out than conditionally cooperate and register under opt-in, as there is less incentive to follow a ‘good shepherd’ compared to a ‘lone-wolf’.

**Table 3:** Conditional Cooperation Within Rounds (Random Effects GLS Models with group level clustered random error) on group averages of decisions to register plus Covariates - the within round Lone-Wolf Effect. The dependent variable is the group average of the Decision to Register (for each individual, 1 = on the register: actively registered under opt-in and not opted-out under opt-out, or 0 = not on the register: not having registered under opt-in and opted-out under opt-out). We have the following independent variables. *Round* is the game number 1–22, *Opt-In:* 0 = Opt-out and = 1 = Opt-in, *Lag Period Decisions* = percent of group registered in the previous period played within the present round. *Sex:* Female = 0, Male = 1. *Game Beliefs:* Not believe it is a game about organ donation = 0, Believe it is a game about organ donation = 1, *Organ Donor Register:* 0 if report not currently registered and 1 if currently registered.

Decision to Register	With Individualistic Feedback			
	Coefficient (SE_robust_)	p	95%	
			Lower	Upper
	Model 5			
Round	−0.002 (0.002)	0.226	−0.006	0.001
Opt-In	**0.427 (0.056)**	**0.000**	**0.317**	**0.538**
Lag Period Decisions	**0.602 (0.060)**	**0.000**	**0.485**	**0.720**
Lag Period Decisions * Opt-In	**−0.181 (0.085)**	**0.033**	**−0.347**	**−0.015**
Age	0.021 (0.026)	0.415	−0.030	0.072
Sex	0.113 (0.112)	0.310	−0.105	0.332
Game Beliefs	0.085 (0.143)	0.551	−0.196	0.367
Organ Donor Register	0.028 (0.159)	0.860	−0.284	0.340
Constant	−0.568 (0.557)	0.308	−1.659	0.523
R^2^_overall_	0.253			
N of Observations(Groups)	1,350 (35)			

**Figure 4:**
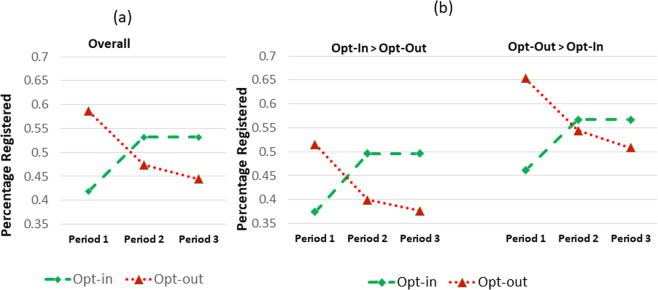
Percentage Registered as a Function of Overall Default Policy (**a**) and Default Policy Change (**b**).

To further compare the effects of ‘lone wolves’ versus ‘good shepherds’ we chart the percentages of participants remaining on the register under opt-out and registering under opt-in in each *period* within a round (Fig. 4a,b) in the presence of individualistic feedback. With individualistic feedback, participants can observe and respond to others actions by changing or maintaining their registration status in the subsequent period. The percentage at each period reflects the numbers switching in their decision to register (under opt-in) or deregister (under opt-out). Overall (Fig. 4a), under opt-out, initially in period 1 a large number deregister (‘lone wolves’), with others also deregistering in subsequent periods 2 and 3. Under opt-in, there is an initial rise (‘good shepherds’) followed by a plateauing out. Relative to the default, these changes are more pronounced for opt-out if moving from opt-in to opt-out, and for opt-in if moving from opt-out to opt-in (Fig. 4b). Thus, over rounds opt-in with individual feedback results in more registered donors.

## Discussion

These results extend our understanding of the dynamics of conditional cooperation and show it is influenced by the nature of the cooperative context (opt-out vs opt-in). When there is immediate individualistic feedback within a round, conditional cooperation is seen immediately. Conditional cooperation across rounds is also observed. However, this pattern is modified by the type of cooperative or free-riding context. When it is a cooperative context (opt-out) and people choose to leave, immediate individualistic feedback enhances negative conditional cooperation resulting in people following suit and leaving (the ‘lone wolf effect’). This effect of conditional cooperation is reversed for a free-riding context (opt-in) with people following a ‘good shepherd’ and cooperating. However, this ‘good shepherd’ effect is not as strong as the ‘lone wolf’ effect. As conditional cooperation is often referred to as a positive force for cooperation^6,8^, we propose that the influence of ‘lone wolves’ within an opt-out context undermines cooperation and represents a *dark side* of conditional cooperation. This is consistent with a growing literature within personality theory showing that many traits believed to be solely positive (e.g., conscientiousness) have a dark side^43,44^ and this literature is also growing in economics [see 39]. This mixture of positive and negative aspects of a trait or preference may help explain why their heterogeneity has an adaptive evolutionary basis^44–46^. Furthermore, the opposing negative ‘lone wolf’ and positive ‘good shepherd’ effects result in comparably lower cooperation levels under opt-out, contrary to the equilibrium predictions of cooperation under opt-out and non-cooperation under opt-in with feedback. Thus, these results clearly identify a boundary condition for the power of the cooperative default – it is maximally powerful in the absence of feedback on others behaviour, and the conditions when lone wolves trigger off the dark side of conditional cooperation that serves to undermine cooperation^39^.

### Defaults mechanism and lone wolf and good shepherd effects

**Figure 5:**
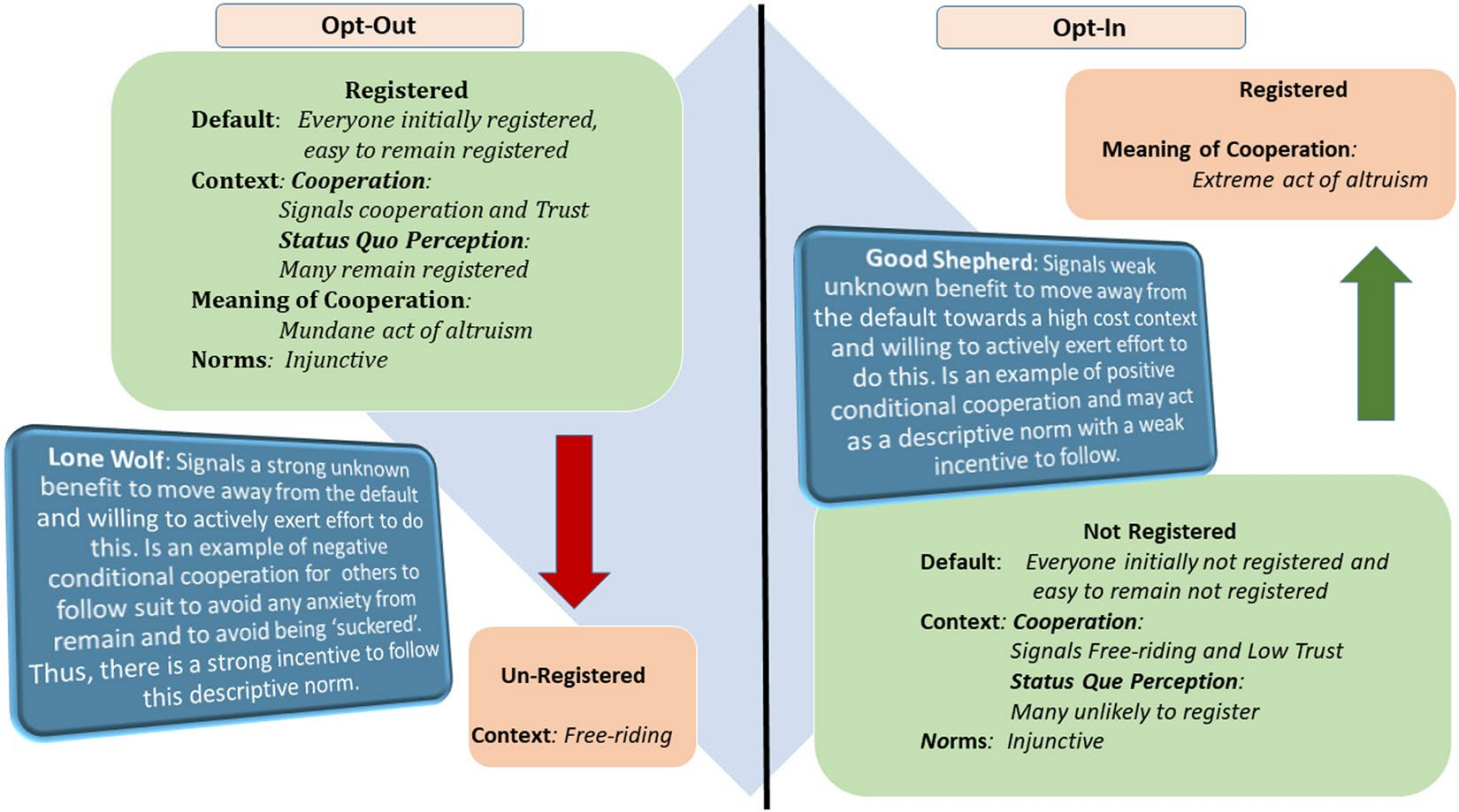
Schematic representation of the processes supporting ‘lone wolf’ and ‘good shepherd’ effects.

Using the three characteristics that support the power of the default (*easy* in reducing the decision maker’s *effort*, *biases* the decision maker’s behaviour towards the status *quo response*, and allows for the *perceived meaning of default* to be inferred) and norm theory^47^ we develop our psychological understanding the ‘lone wolf’ and ‘good shepherd’ effects and why the ‘lone wolf’ effect is stronger (Fig. 5). In both the opt-out and opt-in settings moving from the easy default option signals to others that the person moving must perceive some benefit from leaving the default. The perceived benefit will be determined by the meaning of the cooperative behaviour as a function of the default. In our case, organ donation is perceived as a *mundane* and *everyday* cooperative act under an opt-out and an *extreme act of cooperation* under opt-in^21^. Thus, deregistering under opt-out signals that the person is willing to exert cognitive and physical costs and effort to exit the easy option that everyone is part of. This may signal that the person knows something that others do not. Those who remain registered may experience some anxiety about remaining due to any uncertainty caused by the person deregistering^48^. Under the opt-in default the ‘good shepherd’ is leaving the default to move towards a cooperative act perceived as an extreme act of cooperation and thus costly. Under these conditions people are less likely to follow as they are less likely to perceive that the alternative is more beneficial than the *status quo*. This analysis of the meaning of the cooperative act afforded by the default supports psychologically why the ‘lone wolf’ effect is stronger than the ‘good shepherd’ effect. While we framed our experiment in terms of organ donation, similar arguments can be applied to the use of opt-out policies to encourage vaccination behaviour and environmental conservation^12–14^.

The observed effects may also reflect normative influences (Fig. 5). Defaults are often perceived as indicating what policy makers or the authorities feel that people ought to do^18,49^. As such, people follow the default as it represents an injunctive norm of what people *ought to* do or what is *approved of*. Similarly, observed conditional cooperation may be regarded as a descriptive norm of what people actually do. There is a growing body of work exploring the effects of both types of norm on cooperation^50–59^, with injunctive norms having a stronger effect on cooperation than descriptive norms^52,59^. Our findings show that in fact the dynamic expression of conditional cooperation (descriptive norms) operates as a function of the cooperative default (injunctive norms) setting. This is consistent with *social radar theory* (SRT) of norms which suggests that people utilize the default information in injunctive norms, which like a radar signal they reflect back off others’ actions (descriptive norms) to guide their most strategic future action they can take^47^. Indeed, there is good evidence that norms can be manipulated to influence cooperation^60,61^. For example, moral nudges (e.g., ‘What do you think is the morally right thing to do?’, or ‘What do you think society thinks is the morally right thing to do?’) have been shown to enhance cooperation and could be used to ameliorate the ‘lone wolf’ effect and enhance the ‘good shepherd’ effect^60^. Such ‘nudges’ may be effect to enhance cooperation with respect to behaviours like organ donation under both opt-in and opt-out settings.

### Policy implications

Insofar as our experiment serves as a parable of conditional cooperation in organ donation, via the induced value of incentives relevant to the context, our results also have some implications regarding the growing debate about the effectiveness of the opt-out policies for organ donation. Critics point out that while an opt-out policy for organ donation increases the potential donor pool^5^, the policy results in: (1) a large variance in this rate (e.g., Sweden and Spain have an opt-out system, yet Sweden remains one of the lowest-ranked countries for organ donation in Europe, and Spain the highest)^19^, (2) reduced numbers of living donations^19,62^, (3) increased ethical and attitudinal concerns about ‘State’ ownership of organs^63^, feelings of coercion and lack of autonomy^64^ and (4) uncertainty about the exact meaning of an individuals presumed consent^65^. Our results serve as a warning about a possible negatives effect of an opt-out policy in the age of social media. That is, the overall positive effect of an opt-out policy on organ donation does not take into account the potential effects of feedback (social media posts and updates) on donor status. This is particularly pertinent now in the age of ‘social media’ where individualistic feedback in terms of status updates on organ donation is available^24–28^. Thus, the detrimental effect of the ‘lone wolf’ effect is a very real possibility^29^, and is made worse by spill-over effects when moving to opt-out from opt-in as most countries do^19^. However, harnessing the ‘good shepherd’ effect could help encourage registration under opt-in as exemplified by the ‘Facebook’ effect^25^. Thus, we suggest that healthcare policymakers should consider staying with the opt-in default and investing in strategic nudges (e.g., promoting status updates on Facebook) to increase registration^26^ rather than move to an opt-out policy. If moving to an opt-out policy then strategies to ameliorate the ‘lone wolf’ effect need to be considered. This could involve the use of moral norms to ameliorate the ‘lone wolf’^60^ as well as strategies to monitor social media and ‘fake news’ or negative stories about organ donation should be considered.

### Limitations

While we show ‘lone wolf’ effect is stronger than the ‘good shepherd’ effect, it must be acknowledged that this is in a Western university sample and needs to be replicated cross-culturally^66^. We mainly focused on the strategic choices under incentives induced by the experimental setup. Future research can investigate the influence of other psychological motivations such as kin altruism in familial donations. Furthermore, we examined specifically people’s active decisions to make public their decision to register under opt-in and deregister under opt-out. People may also make these decisions privately or just to close relatives or loved ones. Thus, we model a specific aspect of organ donor registration behaviour, but one that we feel will be of increasing importance in the age of social media and as more countries move to an opt-out policy for organ donation. Indeed, with respect to organ donation losing one or two potential donors due to a ‘lone wolf’ effect would have major significance for those waiting for an organ. Indeed, it has been argued that an effect size should be considered in relative, not absolute, terms^67^ and as such there is likely a real clinical impact of the ‘lone wolf’.

## Data availality

The data files for the economic game reported here will be made available on the OSF page (https://osf.io/2eu3d/) as well as waves 1 and 2 of the online experiment.

## Supplementary information

Supplementary information.
