# Supplementary material for: When Lone Wolf Defectors Undermine the Power of the Opt-Out Default
Source: Sci Rep. 2020 Jun 2;10:8973. doi: 10.1038/s41598-020-65163-1 (PMC7265288; doi:10.1038/s41598-020-65163-1)
Supplement: Supplementary file 1 — Supplementary information. [file 41598_2020_65163_MOESM1_ESM.docx]

**Supplementary Files**

| **File** | **Pages** |
| --- | --- |
| **Supplementary File S1: Survey Experiments: Opt-in and Opt-out Policies Frame Different Cooperative Defaults**  *Method and results from two experiments exploring the perceptions of opt-out and opt-in policies in terms of perceived status quo and trust.* | 2-11 |
| **Supplementary File S2: Game Theoretic Analysis and R Code for Simulations**  *Game theoretic analysis of organ donation*  *Baseline experimental parameter values result in excess organ demand*  *R code for simulated expected payoffs as a function of the number of other registered donors* | 12-23 |
| **Supplementary File S3: Experiment 3: Organ Donation Game – Experimental Design, Instructions, Power and Payments** | 24-47 |
| **Supplementary File S4: Additional Supporting Analyses for Organ Donation Game**  *This file contains detail on our [A] game and experimental design, [B] the instructions given to participants, [C] Game Screen Shots, [D] Earnings Stage Display Error [E] Post-Game Questions, power calculations, and [F] payoff structure* | 48-62 |

**Supplementary File S1: Survey experiment: Opt-in and Opt-out Policies Frame Different Cooperative Defaults**

**Introduction**

The survey experiment examines if for opt-in people have a status quo perception of a few people register (the majority free-rid) and that for the opt-out policy that the status quo perception is that the majority remain registered and cooperate. We further explore the extent to which participants trust people to remain registered under opt-out and become registered under opt-in. policy frames a mindset of free-riding and low trust, and the opt-out policy of cooperation and trust [19]. These are part of a larger set of studies and we report only the variables that are pertinent to this paper. However, we inclide all the measures we used as part of these wider studies to ensure full transparancy.

**Method**

**Samples.** A convenience sample of one hundred participants (*M* age = 24.84; SD = 4.44; 50% female; 54% on the organ donor register) took part in wave 1 and 159 in wave 2 (*M* age = 20.06; SD = 1.90; 52% female; 50% on the organ donor register). These were all University of Nottingham students.

**Procedure and Materials.** Participants in waves 1 and 2 were randomly allocated to consider a description of either an opt-out or opt-in organ donation policy (full verbatim materials used are provide in the appendix to the Supplementary File S1).

***Default Manipulation.*** In *the opt-in* condition participants were told: ‘Healthcare systems in countries around the world adopt different policies to meet the need for donated organs for transplantation. We would like you to consider a country that has adopted an opt-in’ policy. An opt-in policy works as follows. A person is considered automatically, by default, to be a non-donor after their death, but if they wish to be an organ donor after their death, they actively have to indicate, for example, by registering as an organ donor, that they wished to donate their organs after their death.’

In the *opt-out* condition participants were told: ‘Healthcare systems in countries around the world adopt different policies to meet the need for donated organs for transplantation. We would like you to consider a country that has adopted an ‘opt-out’ policy. An opt-out policy works as follows. A person is considered automatically, by default, to be a donor after their death, but if they wish not to donate their organs after their death, they actively have to indicate, for example, by de-registering as an organ donor, that they do not wish to donate their organs after their death.’

***Outcomes***. Participants in the opt-in condition indicated the percentage of the population they think register as donors (status quo perception) and if they think that others can be trusted to register (‘No’ or ‘Yes’). Participants in the opt-out condition, indicated the percentage they think stay registered (status quo perception) as a donor and if they think others can be trusted to stay registered (‘No’ or ‘Yes’).

**Ethics**. Waves 1 and 2 of the survey experiment were approved and registered in accordance with the ethical procedures of the School of Psychology Ethic committee at the University of Nottingham (references 543 & 550 respectively) and were conducted in accordance with the relevant guidelines and regulations of the British Psychological Society (<https://www.bps.org.uk/sites/bps.org.uk/files/Policy/Policy%20-%20Files/BPS%20Code%20of%20Ethics%20and%20Conduct%20%28Updated%20July%202018%29.pdf>) and the University of Nottingham (<https://workspace.nottingham.ac.uk/display/ResEth/Code+of+Research+Conduct+and+Research+Ethics?preview=/123507321/298524330/Code%20of%20Research%20Conduct%20and%20Research%20Ethics%20(Version%206a)%20(revisions%20Mar%202019).docx>). Participants in experiments 1 and 2 provided signed informed consent with their anonymity assured.

**Results & Discussion**

Consistent with our argument, participants in both experiments indicated that a significantly greater percentage of the population would be registered under opt-out than opt-in (73.1% _opt-out_, *SD* = 14.95 [N = 50] vs 29.8% _opt-in_, *SD* = 21.33 [N = 49], Z = 7.33, p = .000 in experiment 1 and 72.6% _opt-out_, *SD* = 21.05 [N = 80] vs 32.8% _opt-in_, *SD* = 21 [N = 77].18, Z = 8.58, p = .000 in experiment 2). Participants were also more likely to *trust* others to remain as an organ donor under opt-out than to register under opt-in (89% _opt-out_ vs 42% _opt-in_, OR = 12.5, *p* = .000; *95%CI* = 4.20, 36.98 [N = 97] experiment 1 and 86% _opt-out_ vs 52% _opt-in_, OR = 5.80, *p* = .000; *95%CI* = 2.66, 12.62 experiment 2 [N = 157]).

Thus, the opt-out default is seen as one where by a large number of people are trusted to remain as donor and the opt-in as one where a few people are trusted to register.

**Experiment 1. Full Materials**

***The Opt-In Default System for Diseased Organ Donation:*** Healthcare systems in countries around the world adopt different policies to meet the need for donated organs for transplantation. We would like you to consider a country that has adopted an **‘opt-in’** policy. An **opt-in** policy works as follows.

1. A person is considered automatically, by default, to be a **non-donor** after their death, but
2. If they wish to be an organ donor after their death, they actively have to indicate, for example, **by registering as an organ donor**, that they wished to donate their organs after their death.

To check you understand what an opt-in system is please answer the following question.

1. Under an opt-in system people are automatically considered to be a non-donor and have to register if they wish to be an organ donor? True : False

**Considering an opt-in system**, please answer the following questions.

1. What percentage of the population do you think are registered to be a donor under opt-in? ____%
2. Would you register under an opt-in system? No : Yes
3. Do you think an opt-in system is fair? No : Yes
4. Do you think others can be trusted to register as a donor under opt-in? No : Yes

**Living Donations**: People can also make living donations. That is, while alive people can donate a kidney, a lobe of their liver or join the register to donate bone marrow to a stranger.

Considering an **opt-in system**, as described above, please indicate your response from 1 (Not at all likely) to 7 (Extremely likely) to each question below

|  | Not at all likely |  |  |  |  |  | Extremely likely |
| --- | --- | --- | --- | --- | --- | --- | --- |
| **Under an opt-in system to what extent you would be willing to:** |  |  |  |  |  |  |  |
| Donate a kidney to a stranger | 1 | 2 | 3 | 4 | 5 | 6 | 7 |
| Donate a kidney to a relative | 1 | 2 | 3 | 4 | 5 | 6 | 7 |
| Donate a lobe of your liver to a stranger | 1 | 2 | 3 | 4 | 5 | 6 | 7 |
| Donate a lobe of your liver to a relative | 1 | 2 | 3 | 4 | 5 | 6 | 7 |
| Donate bone marrow | 1 | 2 | 3 | 4 | 5 | 6 | 7 |
| Donate 20% of your salary to charity | 1 | 2 | 3 | 4 | 5 | 6 | 7 |
| Volunteer your time regularly to help out at a local charity shop | 1 | 2 | 3 | 4 | 5 | 6 | 7 |
| Give someone your car parking ticket, for free, when there is still time left on it | 1 | 2 | 3 | 4 | 5 | 6 | 7 |
| Vote in a general election | 1 | 2 | 3 | 4 | 5 | 6 | 7 |
| Donate your unwanted clothes to charity | 1 | 2 | 3 | 4 | 5 | 6 | 7 |
| Donate any unwanted toys to charity | 1 | 2 | 3 | 4 | 5 | 6 | 7 |
| Sign a petition | 1 | 2 | 3 | 4 | 5 | 6 | 7 |
| Donate blood once |  |  |  |  |  |  |  |
| Donate blood regularly |  |  |  |  |  |  |  |
| Give someone a concert ticket for free that you can no longer use | 1 | 2 | 3 | 4 | 5 | 6 | 7 |

1. Have you signed on the organ donor register? Yes : No
2. Have you ever donated blood? Yes : No;
3. Are you currently an active blood donor? Yes : No
4. Have you donated blood in the past but do not anymore? Yes : No
5. How old are you? ________ What is your sex: Male : Female

***The Opt-Out Default System for Diseased Organ Donation:*** Healthcare systems in countries around the world adopt different policies to meet the need for donated organs for transplantation. We would like you to consider a country that has adopted an **‘opt-out’** policy. An **opt-out** policy works as follows.

1. A person is considered automatically, by default, to be a **donor** after their death, but
2. If they wish **not to donate their** organs after their death, they actively have to indicate, for example, **by de-registering as an organ donor**, that they do not wished to donate their organs after their death.

To check you understand what an opt-out system is please answer the following question.

1. Under an opt-out system people are automatically considered to be a donor and have to de-register if they wish not to be an organ donor? True : False

**Considering an opt-out system**, please answer the following questions.

1. What percentage of the population do you think stay registered to be a donor under an opt-out system? ________%
2. Would you stay registered under an opt-out system? No : Yes
3. Do you think an opt-out system is fair? No : Yes
4. Do you think others can be trusted to stay registered as a donor under an opt-out system?

No : Yes

**Living Donations**: People can also make living donations. That is, while alive people can donate a kidney, a lobe of their liver or join the register to donate bone marrow to a stranger.

Considering an opt-out system, as described above, please indicate your response from 1 (Not at all likely) to 7 (Extremely likely) to each question below.

|  | Not at all likely |  |  |  |  |  | Extremely likely |
| --- | --- | --- | --- | --- | --- | --- | --- |
| **Under an opt-out system to what extent you would be willing to:** |  |  |  |  |  |  |  |
| Donate a kidney to a stranger | 1 | 2 | 3 | 4 | 5 | 6 | 7 |
| Donate a kidney to a relative | 1 | 2 | 3 | 4 | 5 | 6 | 7 |
| Donate a lobe of your liver to a stranger | 1 | 2 | 3 | 4 | 5 | 6 | 7 |
| Donate a lobe of your liver to a relative | 1 | 2 | 3 | 4 | 5 | 6 | 7 |
| Donate bone marrow | 1 | 2 | 3 | 4 | 5 | 6 | 7 |
| Donate 20% of your salary to charity | 1 | 2 | 3 | 4 | 5 | 6 | 7 |
| Volunteer your time regularly to help out at a local charity shop | 1 | 2 | 3 | 4 | 5 | 6 | 7 |
| Give someone your car parking ticket, for free, when there is still time left on it | 1 | 2 | 3 | 4 | 5 | 6 | 7 |
| Vote in a general election | 1 | 2 | 3 | 4 | 5 | 6 | 7 |
| Donate your unwanted clothes to charity | 1 | 2 | 3 | 4 | 5 | 6 | 7 |
| Donate any unwanted toys to charity | 1 | 2 | 3 | 4 | 5 | 6 | 7 |
| Sign a petition | 1 | 2 | 3 | 4 | 5 | 6 | 7 |
| Donate blood once |  |  |  |  |  |  |  |
| Donate blood regularly |  |  |  |  |  |  |  |

1. Have you signed on the organ donor register? Yes : No
2. Have you ever donated blood? Yes : No;
3. Are you currently an active blood donor? Yes : No
4. Have you donated blood in the past but do not anymore? Yes : No
5. How old are you? ________ What is your sex: Male : Female

**Experiment 2: Full Materials**

***The Opt-In Default System for Diseased Organ Donation:*** Healthcare systems in countries around the world adopt different policies to meet the need for donated organs for transplantation. We would like you to consider a country that has adopted an **‘opt-in’** policy. An **opt-in** policy works as follows.

1. A person is considered automatically, by default, to be a **non-donor** after their death, but
2. If they wish to be an organ donor after their death they have to actively indicate, for example, **by registering as an organ donor**, that they wished to donate their organs after their death.

To check you understand what an opt-in system is please answer the following question.

1. Under an opt-in system people are automatically considered to be a non-donor and have to register if they wish to be an organ donor? True : False

**Considering an opt-in system**, please answer the following questions.

1. What percentage of the population do you think are registered to be a donor under opt-in? ____%
2. Would you register under an opt-in system? Yes : No
3. Do you think an opt-in system is fair? Yes : No
4. Do you think others can be trusted to register as a donor under opt-in? Yes : No

**Living Donations**: People can also make living donations. That is, while alive people can donate a kidney, a lobe of their liver or join the register to donate bone marrow to a stranger.

Considering an **opt-in system**, as described above, please indicate your response from 1 (Not at all likely) to 7 (Extremely likely) to each question below

|  | Not at all likely |  |  |  |  |  | Extremely likely |
| --- | --- | --- | --- | --- | --- | --- | --- |
| **Under an opt-in system to what extent you would be willing to:** |  |  |  |  |  |  |  |
| Donate a kidney to a stranger | 1 | 2 | 3 | 4 | 5 | 6 | 7 |
| Donate a kidney to a relative | 1 | 2 | 3 | 4 | 5 | 6 | 7 |
| Donate a lobe of your liver to a stranger | 1 | 2 | 3 | 4 | 5 | 6 | 7 |
| Donate a lobe of your liver to a relative | 1 | 2 | 3 | 4 | 5 | 6 | 7 |
| Donate bone marrow | 1 | 2 | 3 | 4 | 5 | 6 | 7 |

Considering an **opt-in system**, as described above image you are at a social event and start talking to a new person please rate how attractive, compassionate, intelligent and well educated they are where 1 = not at all and 10 = extremely. Please insert a number in each cell below:

|  | **Attractive** | **Compassionate** | **Intelligent** | **Well Educated** |
| --- | --- | --- | --- | --- |
| A **man** who tells you he has registered as an organ donor |  |  |  |  |
| A **woman** who tells you she has registered as an organ donor |  |  |  |  |
| A **man** who tells you he has donated a kidney to a relative |  |  |  |  |
| A **woman** who tells you she has donated a kidney to a relative |  |  |  |  |
| A **man** who tells you he would not register as an organ donor |  |  |  |  |
| A **woman** who tells you she would not register as an organ donor |  |  |  |  |

The following statements inquire about your thoughts and feelings in a variety of situations. For each item, indicate how well it describes you by circling the appropriate number on the scale. Answer as honestly as you can.

|  | **Does not describe me very well** | |  |  |  | **Describes me very well** | |
| --- | --- | --- | --- | --- | --- | --- | --- |
| I often have tender, concerned feelings for people less fortunate than me | 1 | 2 | 3 | 4 | 5 | 6 | 7 |
| When I see someone being taken advantage of, I feel kind of protective towards them | 1 | 2 | 3 | 4 | 5 | 6 | 7 |
| I am often quite touched by things I see happen | 1 | 2 | 3 | 4 | 5 | 6 | 7 |
| I would describe myself as a pretty soft-hearted person | 1 | 2 | 3 | 4 | 5 | 6 | 7 |

1. Have you signed on the organ donor register? Yes : No
2. Are you willing to donate an organ? Yes : No
3. Have you ever donated blood? Yes : No;
4. Are you currently an active blood donor? Yes : No
5. Have you donated blood in the past but do not anymore? Yes : No

The following, are questions about your personal background. You should attempt to fill in all the answers. However, if you prefer not to, or cannot answer a particular question, please leave it blank.

Age: ______, Sex: Male Female, Are you currently in a relationship: Yes No

What is your sexual orientation? a) Heterosexual, b) Homosexual, c) Bisexual (*Please circle as appropriate).*

***The Opt-Out Default System for Diseased Organ Donation:*** Healthcare systems in countries around the world adopt different policies to meet the need for donated organs for transplantation. We would like you to consider a country that has adopted an **‘opt-out’** policy. An **opt-out** policy works as follows.

1. A person is considered automatically, by default, to be a **donor** after their death, but
2. If they wish **not to donate their** organs after their death they have to actively indicate, for example, **by de-registering as an organ donor**, that they do not wished to donate their organs after their death.

To check you understand what an opt-in system is please answer the following question.

1. Under an opt-out system people are automatically considered to be a donor and have to de-register if they wish not to be an organ donor? True : False

**Considering an opt-out system**, please answer the following questions.

1. What percentage of the population do you think stay registered to be a donor under an opt-out system? ________%
2. Would you stay registered under an opt-out system? No : Yes
3. Do you think an opt-out system is fair? No : Yes
4. Do you think others can be trusted to stay registered as a donor under an opt-out system? No : Yes

**Living Donations**: People can also make living donations. That is, while alive people can donate a kidney, a lobe of their liver or join the register to donate bone marrow to a stranger.

Considering an opt-out system, as described above, please indicate your response from 1 (Not at all likely) to 7 (Extremely likely) to each question below.

|  | Not at all likely |  |  |  |  |  | Extremely likely |
| --- | --- | --- | --- | --- | --- | --- | --- |
| **Under an opt-out system to what extent you would be willing to:** |  |  |  |  |  |  |  |
| Donate a kidney to a stranger | 1 | 2 | 3 | 4 | 5 | 6 | 7 |
| Donate a kidney to a relative | 1 | 2 | 3 | 4 | 5 | 6 | 7 |
| Donate a lobe of your liver to a stranger | 1 | 2 | 3 | 4 | 5 | 6 | 7 |
| Donate a lobe of your liver to a relative | 1 | 2 | 3 | 4 | 5 | 6 | 7 |
| Donate bone marrow | 1 | 2 | 3 | 4 | 5 | 6 | 7 |

Considering an **opt-out system**, as described above image you are at a social event and start talking to a new person please rate how attractive, compassionate, intelligent and well educated they are where 1 = not at all and 10 = extremely. Please insert a number in each cell below

|  | **Attractive** | **Compassionate** | **Intelligent** | **Well Educated** |
| --- | --- | --- | --- | --- |
| A **man** who tells you he has remained as an organ donor |  |  |  |  |
| A **woman** who tells you she has remained as an organ donor |  |  |  |  |
| A **man** who tells you he has donated a kidney to a relative |  |  |  |  |
| A **woman** who tells you she has donated a kidney to a relative |  |  |  |  |
| A **man** who tells you he opted-out of organ donor register |  |  |  |  |
| A **woman** who tells you she opted-out of organ donor register |  |  |  |  |

The following statements inquire about your thoughts and feelings in a variety of situations. For each item, indicate how well it describes you by circling the appropriate number on the scale. Answer as honestly as you can.

|  | **Does not describe me very well** | |  |  |  | **Describes me very well** | |
| --- | --- | --- | --- | --- | --- | --- | --- |
| I often have tender, concerned feelings for people less fortunate than me | 1 | 2 | 3 | 4 | 5 | 6 | 7 |
| When I see someone being taken advantage of, I feel kind of protective towards them | 1 | 2 | 3 | 4 | 5 | 6 | 7 |
| I am often quite touched by things I see happen | 1 | 2 | 3 | 4 | 5 | 6 | 7 |
| I would describe myself as a pretty soft-hearted person | 1 | 2 | 3 | 4 | 5 | 6 | 7 |

1. Have you signed on the organ donor register? Yes : No
2. Are you willing to donate an organ? Yes : No
3. Have you ever donated blood? Yes : No
4. Are you currently an active blood donor? Yes : No
5. Have you donated blood in the past but do not anymore? Yes No

The following, are questions about your personal background. You should attempt to fill in all the answers. However, if you prefer not to, or cannot answer a particular question, please leave it blank.

Age: ______, Sex: Male Female, Are you currently in a relationship: Yes No

What is your sexual orientation? a) Heterosexual, b) Homosexual, c) Bisexual (*Please circle as appropriate).*

**Supplementary File S2: Game Theoretic Analysis of Organ Donation and R Code for Simulations**

The organ donation game has three players indexed by *i* = 1, 2, 3. It involves a *choice task* where players choose their donor registration status, followed by an *earnings stage* where players realize their health outcomes. Choice tasks and earnings stages each contain a number of periods. The choice task starts with a default action *d* that is exogenously set at *d* = 0 under opt-in and *d* = 1 under opt-out. Players choose between being unregistered or registered on the organ donor list. We denote these actions as *a_i_* = 0 or *a_i_* = 1, respectively. Following Kessler and Roth (2012), each player can change from the default once per game. Being a registered donor incurs a psychological cost *c* = 0.4, which reflects psychological costs such as the ‘fear of worse medical treatment or discomfort […] from thinking about his own death.’ The total number of registered co-players is *n_-i_* = *a*_1_ + *a*_2_ + *a*_3_ – *a_i_*. The state-of-play under some default is described by (*d*: *a_i_*, *n_-i_*). Games start at *initial state* (0: 0, 0) under opt-in and (1: 1, 2) under opt-out.

In the game of Kessler and Roth (2012), there is no individualistic feedback and the choice task involves only one period in which players decide simultaneously. Feedback is provided only at the end of the choice task and therefore cannot influence decisions in the same game. We extend their game to study the opt-out default and individualistic feedback in multi-period choice tasks. Following the dynamic voluntary contributions game of Tan et al. (2015), updates on the state-of-play are provided whenever a co-player changes from the default, before the game continues on to the next period. A choice task ends when no player can or wants to change registration status. Individualistic feedback is received in the interim and can influence decisions in the present game. The choice task in such games last at least one period (if no player changes or all players change in the first period) and at most three periods (if one player changes in each period).

After all players have decided, they experience their health outcomes in the *earnings stage*. In our experiment, for each period that a player’s organs are fully functioning, she will earn 1 point and the stage continues to the next period. If one’s brain fails, then the player will lose 1 point and the game ends in that period. If one’s brain is still active but kidneys fail, then one can continue for up to five periods without active kidneys while waiting for a donor kidney. Before receiving a donor kidney, one will earn 0 points. A player can receive a donor kidney from a co-player who is a donor whose brain has failed but kidneys have not failed before. If in one of these periods this player receives a kidney from someone else, then she will start earning points again. Once a kidney has been donated, it cannot be donated again. If a kidney is not received within those five periods, the player will lose 1 point and the game ends in the last of those five periods.

**Table A1.** *Payoff structure: expected payoffs as a function of number of other registered donors in the group for our experiment*

|  | 2 others in  *a_-i_* = 2 | 1 other in  *a_-i_* = 1 | 0 others in  *a_-i_* = 0 |
| --- | --- | --- | --- |
| I’m in *a_i_* = 1 | 4.36 | 4.14 | 3.87 |
| I’m out *a_i_* = 0 | 4.75 | 4.56 | 4.26 |

Organ donation incentives are described by *v* = *π*(*n_-i_*) – *c*, which is the expected benefit as a function of the number of other registered donors net of cost. The next section shows that our chosen baseline experimental parameter values result in excess organ demand, which is externally valid. Table A1 shows the net expected payoffs simulated with our experimental parameter values. The computation was performed with the software R (see codes below). Net expected payoffs strictly increase with the number of registered co-players, and decrease with being registered. We can analyze strategic choices with this simplified payoff structure.

**Lemma 0 (opt-in or opt-out without individualistic feedback):** In games without individualistic feedback with *c_i_* > 0, player *i* chooses *a_i_* = 0. This is the unique equilibrium.

**Proof:** Player *i* cannot influence *n_-i_*, therefore the possible benefits from receiving an organ is independent of her decision, which is in turn determined only by cost *c*.

In games without feedback, *a_i_* = 1 is a strictly dominated strategy if *c* > 0 as it decreases one’s payoff by *c* regardless of others’ actions. If *c* > 0 and *d* = 0 (*d* = 1), then one is better off maintaining at (changing to) *a_i_* = 0. Thus *a_i_* = 0 is always a mutual best response. In games without feedback, the Nash equilibrium is *a_i_* = 0 for all defaults.

**Lemma 1 (opt-in with individualistic feedback):** In opt-in games with individualistic feedback with *c_i_* > 0, all players choose *a_i_* = 0 in all periods. This is the unique subgame perfect equilibrium.

**Proof:** Assume in some period, *n_-i_* = 2, then all co-players cannot change their choices. In a state (0: 0, 2), *i* is better off choosing *a_i_* = 0. Now assume *n_-i_* = 1 in some period. Without loss of generality we can assume *a*_3_ = 0. If player 1 then chooses *a*_1_ = 0 and player 2 chooses *a*_2_ = 1 giving *n_-i_* = 2, player 1 will still be better off choosing *a*_1_ = 0. Therefore, player 2 is better off not opting in, and the same is true for player 1. If *n_-i_* = 0, no player will opt in because none of their co-players will follow. Also, if player 1 expects one or both co-players to opt in in the same period she is better off not opting in. Therefore, no player will opt in.

We can also graphically illustrate strategic reasoning in games with individualistic feedback. Let us define the *terminal state* as a state where the game ends because all players have changed their registration status, i.e. (1: 0, 0) in the opt-in game and (1: 1, 2) in the opt-out game. Our backward induction shall start from the terminal state of each game, through paths that ‘backtrack’ to the initial state where the game originates. Figure A1 graphically presents the paths for each game.

**Figure A1.** *Paths from initial states through to terminal states for opt-in (left) and opt-out (right) with individualistic feedback*

***
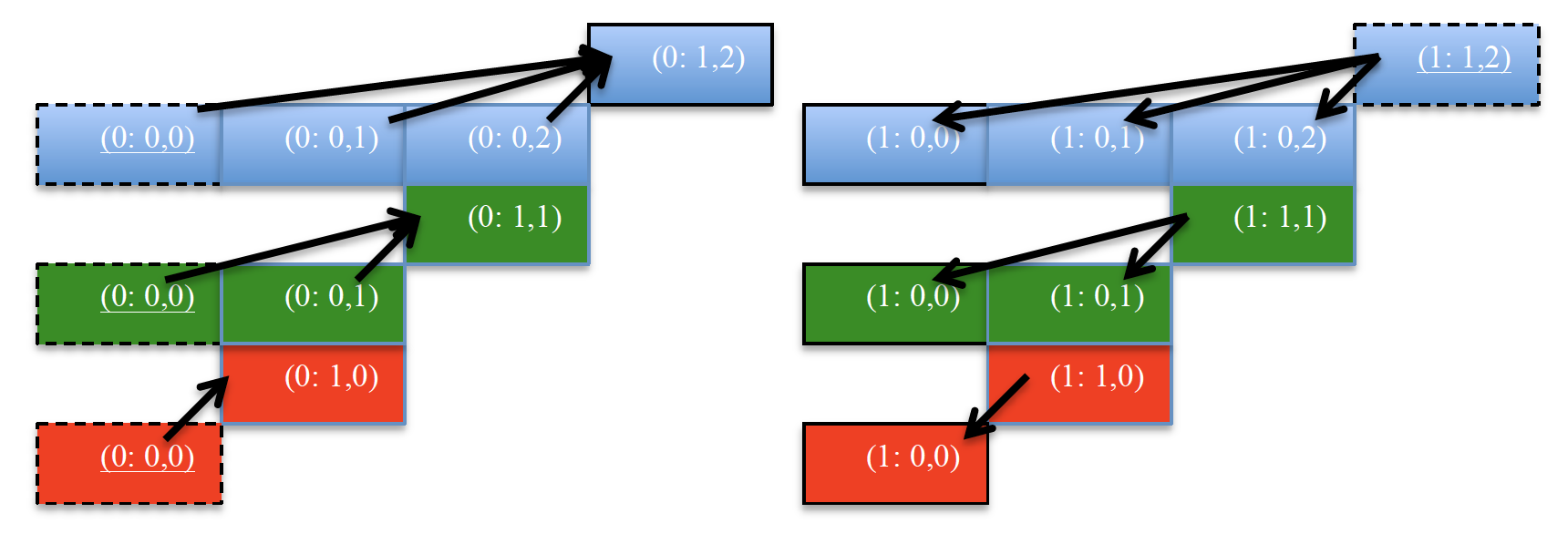
***

***Note.*** Arrows point from preceding states into reachable states. Shortest arrows represent closest preceding states. Different colours represent different players. Adjacent states are symmetric from the non-mover’s perspective. Initial states have dotted borders. Terminal states have thick borders. Solutions are underscored.

In the opt-in game with individualistic feedback, we backtrack from the terminal state (0: 1, 2), which is reachable from (0: 0, 0), (0: 0, 1), and (0: 0,2). Iteratively, one lands on the closest preceding state (0: 0, 2) when her co-players have opted in and reached (0: 1, 1) from (0: 0, 0) or (0: 0, 1).^[[1]](#footnote-1)^ In turn, one lands on the closest preceding state (0: 0, 1) when her co-player has opted in and reached (0: 1, 0) from the initial state (0: 0, 0). Backward inducing from the terminal state (0: 1, 2), opting in results in inferior states and is therefore dominated by not opting in, i.e. staying in their closest preceding states as because *π*(*a_-i_*) – c < *π*(*a_-i_*), which keeps us at the initial state (0: 0, 0). Therefore, players prefer to maintain *a_i_* = 0, and by symmetry the solution is the subgame perfect equilibrium (0: 0, 0).

Having established that opting in is never a best response to others opting in, by backward induction there is no incentive to opt-in as leader in stage 1 or as follower after observing others register. This implies that it does not pay to be a ‘good shepherd’, which we define as players who move off the equilibrium path by unilaterally opting into the registry to lead-by-example, i.e. by changing from the default *a_i_* = 0 to *a_i_* = 1 since no co-player will follow in equilibrium.

**Lemma 2 (opt-out with individualistic feedback):** (i) In the opt-out game with *c_i_* > 0, all players choosing *a_i_* = 0 in all periods is a subgame perfect equilibrium.

(ii) If *π*(0) < *π*(1) – *c* then there are three subgame perfect equilibria where one player opts out immediately and the other two players opt out if and only if *n_-i_* = 0.

(iii) If *π*(0) < *π*(2) – *c*, then there is a subgame perfect equilibrium where all players opt out if and only if *n_-i_* < 2.

**Proof:** (i) After the first period, every player is faced with *n_-i_* = 0 which cannot be changed. Therefore, it is optimal for player *i* to also choose *n_-i_* = 0.

(ii) If, in some period, *n_-i_* = 0 is reached then player *i* chooses *a_i_* = 0. If, however, *n_-i_* = 1, then player *i* with *a_i_* = 1 will weigh the consequences of choosing *a_i_* = 0 or remaining at *a_i_* = 1. If she chooses *a_i_* = 0, then the co-player *k* with *a_k_* = 1 will choose *a_k_* = 0 in the next period and player *i* ends up earning *v_i_* = *π*(0). If co-player *k* does not switch if player *i* did not switch then player *i* earns *v_i_* = *π*(1) – *c*. Therefore, if *π*(0) < *π*(1) – *c*, then both players *i* and *k* are better off if they do not opt out. This implies the following subgame perfect equilibrium strategies: Player *j* opts out in the first period. Player *i* (player *k*) opts out if and only if *n_-i_* = 0 (*n_-k_* = 0). No player can be cheated because she can still opt out in the next period. Note, however, that in states where one player has opted out it is also an equilibrium if both other players opt out simultaneously in the next period. This brings us to the case (iii).

(iii) Let us assume that in states with *n_-i_* = 0 or *n_-i_* = 1 player *i* chooses *a_i_* = 0. According to (ii) this is equilibrium behaviour. In states with *n_-i_* = 2 and *a_i_* = 1, player *i* has to weigh her options. If she chooses *a_i_* = 0, she will earn *v_i_* = *π*(0). If the co-players do not opt out under *n_-i_* = 2 then *a_i_* = 1 will yield *v_i_* = *π*(2) – *c*. Therefore, if *π*(0) < *π*(2) – *c*, then there is a subgame perfect equilibrium where all players remain registered if *n_-i_* = 2 and opt out if *n_-i_* < 2.

If *π*(1) – *π*(2) > *c* and *π*(0) < *π*(1)-*c* then every player would prefer to be the opting-out player from case (ii). For 2(*π*(1) – *π*(2)) > *c* the equilibria from (ii) are efficient, i.e. maximizing aggregate income. But it is difficult to coordinate on one of the equilibria from 2. In our experiments (Table A1), we have *π*(0) > *π*(1) – *c* and *π*(0) > *π*(2) – *c*, i.e. only the two equilibria under (i) and (iii) apply. However, (i) is weakly dominated and hence cannot be sustained in a trembling-hand perfect equilibrium, while (iii) is Pareto-superior and a player is safeguarded from losses if the others opt out, as she can follow suit.

Referring once again the Figure A1, in the opt-out game with individualistic feedback, we backtrack from the terminal state (1: 0, 0), which is reachable from (1: 1, 2), (0: 1, 1), and (0: 1, 0). Iteratively, one lands on the closest preceding state (1: 1, 0) when her co-players have reached (1: 0, 1) from (1: 1, 2) and (1: 1, 1). In turn, one lands on the closest preceding state (1: 1, 1) when her co-player has reached (1: 0, 2) from the initial state (1: 1, 2). The payoff in the terminal state is preferred to those of all states reachable from the initial state as *π*(*a_-i_*) – *c* < *π*(*a_-i_*), but *a_i_* = 1 (to maintain the initial state) dominates *a_i_* = 0 (reaching the terminal state) as *π*(*a_-i_*) – *c* > 0. Therefore, players prefer to maintain *a_i_* = 1 at the start, and by symmetry the solution is the subgame perfect equilibrium (1: 1, 2).

The opt-out subgame perfect equilibrium, therefore, involves a two-part strategy profile where 1) a player should not deviate (i.e. opt-out) from an equilibrium state that is Pareto superior to other feasible equilibrium states that result from players opting out, and 2) if a player is not in such a state because others have opted out, then it pays off to opt-out and move to the next feasible equilibrium state. Backward induction implies that it does not pay to be a ‘lone wolf’ by changing from the default because others will follow in equilibrium.

Because following is not a best response under opt-in but it is a best response under opt-out, we should expect fewer players – if any – to follow ‘good shepherds’ than to follow ‘lone wolves’. We define ‘lone wolves’ as players who move off the equilibrium path by unilaterally opting out from the default before others are observed to free ride, which is attributable to negative spillover effects. Therefore, strategic reasoning suffices to support the hypothesis that the ‘lone wolf’ effect dominates the ‘good shepherd’ effect. The Pareto efficient prediction of the opt-out game is vulnerable to deviations (off-equilibrium defection) by the ‘lone wolf’.

Further, Tan et al. (2015) showed that with a mixture of players motivated by self-interest or inequity aversion (Fehr & Schmidt, 1999), there are incentives to lead and follow in the spirit of conditional cooperation in equilibrium. The underlying intuition is that with incomplete information on co-player types, ‘good shepherds’ lead if there is a sufficiently high probability that they will be followed. However, we also observe ‘lone wolves’ who deviate from the equilibrium even though it does not pay off, suggesting the negative spillover effects and the presence of players with a strong preference for being unregistered. This triggers a cascade of other opt-outs, which is a best response in our equilibrium analysis.

**Baseline experimental parameter values result in excess organ demand**

Our incentive structure largely follows Kessler and Roth (2012). The payoffs due to a player’s health outcomes is normalized to 0 when the player requires a donor organ but no organ is available, and is *v* > 0 when the player requires a donor organ and receives one. A player’s expected payoff π(n_-i_) is a function of the number of co-players who are registered. In the earnings stage, there are three possible health conditions. The first is one where there is a probability of *β* > 0 that one’s brain has failed, which allows the player to donate her kidneys if she is on the register. The second is one where there is a probability of *θ* > 0 that one’s kidneys will fail, and we assume that both kidneys fail at the same time. The third is one where a player’s brains and kidneys are active in a period, which occurs with complementary probability 1 − *θ* − *β* ≥ 0.

When a player’s kidneys fail, she receives a donor kidney if one is available. There are α kidneys made available whenever a co-player dies from brain failure. The probability of getting a donor kidney is determined by how many co-players have registered as donors. In Kessler and Roth’s baseline case, available donor organs are assigned randomly to anyone who needs one. At any one time we expect a *θ* share of players to need organs and a share *β* of players die of brain failure can donate α kidneys each and if they are registered donors.

For benchmarking, let the share of registered donors be *x*. The probability of receiving an organ when needed is *p* = min{*αβ*/*θx*, 1}, implying excess demand for organs when *θ* > *αβx*, which is satisfied by our parameter values *α* = 2, *β* > .10, *θ* > .15. Under opt-in without interventions (e.g. priority schemes), there is no incentive to register if *c* > 0. This is satisfied in our experiment as pecuniary costs are *c* = 0.4 for all players.^[[2]](#footnote-2)^ With this zero-registration prediction as benchmark, observed behavior reveals that an individual’s psychological benefit exceeds cost. These include psychological benefits of altruism or feeling a warm glow by being on the donor register list, versus disutility due to the ‘ick’ factor. The converse holds when people opt-out under an intervention where full registration is in equilibrium.

**R code for simulated expected payoffs as a function of the number of other registered donors**

EARNINGS <- data.frame("P1" = c(0), "P2" = c(0), "P3" = c(0))

donors<-0

donor <- c(0,0,0)

donations<-0

for(i in 1:10000)

{

##

dead <- c(0,0,0)

wait <- c(0,0,0)

random_A <- c(0,0,0)

random_B <- c(0,0,0)

fail_A <- 0.1

fail_B <- 0.15

A <- c(1,1,1)

B <- c(2,2,2)

earnings <- c(2,2,2)

earnings <- earnings - donor*0.4

while(sum(dead)<3)

{

if(sum(B==0)>0)

{

for (i in 1:3)

{

wait[i] <- ifelse (B[i] == 0 & dead[i] == 0, wait[i]+1,wait[i])

if(wait[i] > 5 & dead[i] == 0)

{

dead[i] <- 1

earnings[i]<- earnings[i] - 1

}

}

}

random_A <- runif(3) <0.1

random_A

random_B <- runif(3) <0.15

random_B

donated <- 0

if(sum(random_A>0))

{

for (i in 1:3)

{

if(isTRUE(random_A[i]) & dead[i]==0)

{

dead[i] <- 1

donated <- ifelse(donor[i] == 1 & B[i]==2, donated + 2, donated)

A[i] <- 0

earnings[i]<- earnings[i] - 1

}

}

}

if(sum(random_B>0))

{

for (i in 1:3)

{

if(isTRUE(random_B[i]))

{

B[i] <- ifelse(B[i]>0,0,B[i])

}

}

}

if (sum(B==0) >0 & donated>0)

{

for(i in 1:3)

{

if(wait[i]>0)

{

wait[i] <- 0

B[i] <- 1

donations<-donations + 1

}

}

}

for(i in 1:3)

{

if(dead[i] == 0 & A[i] == 1 & B[i] > 0)

{

earnings[i] <- earnings[i] + 1

}

}

print(c("donated",donated))

print(c("wait",wait))

print(c("random_A",random_A))

print(c("A",A))

print(c("random_B",random_B))

print(c("B",B))

print(c("earnings",earnings))

print("---------------------")

}

EARNINGS <- rbind(EARNINGS,earnings)

}

fname <- paste("Earnings",donors,sep = "_")

write.xlsx(x = EARNINGS, file = paste(fname,".xlsx"), sheetName = "data3", row.names = FALSE )

**Supplementary File S3: Experiment 3: Organ Donation Game – Experimental Design, Instructions, Power and Payments**

This file contains detail on [A] our game and experimental design, [B] the instructions given to participants, [C] Game Screen Shots, [D] Post Game Questions [E] power calculations and [F] payoff structure

**[A] Organ Donor game and experimental design**

**The Organ Donor Game**

***Overview***: The game we developed is based on and developed from the basic game of Kessler and Roth (2012). Our game differs in that we (1) explore policy (opt-in and opt-out) changes and (2) feedback, as well as (3) separate registration choices from *health outcomes* (i.e. whether or not subjects actually donate or need and receive an organ). That is, in the Kessler and Roth game participants can choose to register or not and then they find out if they need organs or have died and can donate. After that, participants play the next game where they make registration choices again. Thus, those who have experienced life or death as a donor or recipient or otherwise in a previous round make living decisions anew. Their learning patterns and behaviour are therefore potentially influenced by their experiences of health outcomes as donor or recipient, which do not feature in reality. To avoid this, we have an experiment where participants make registration choices for a number of *choice tasks* to model how people may change their mind over time, after which they move find out their health outcomes in corresponding *earnings stages*. A schematic of the experimental design is given below.


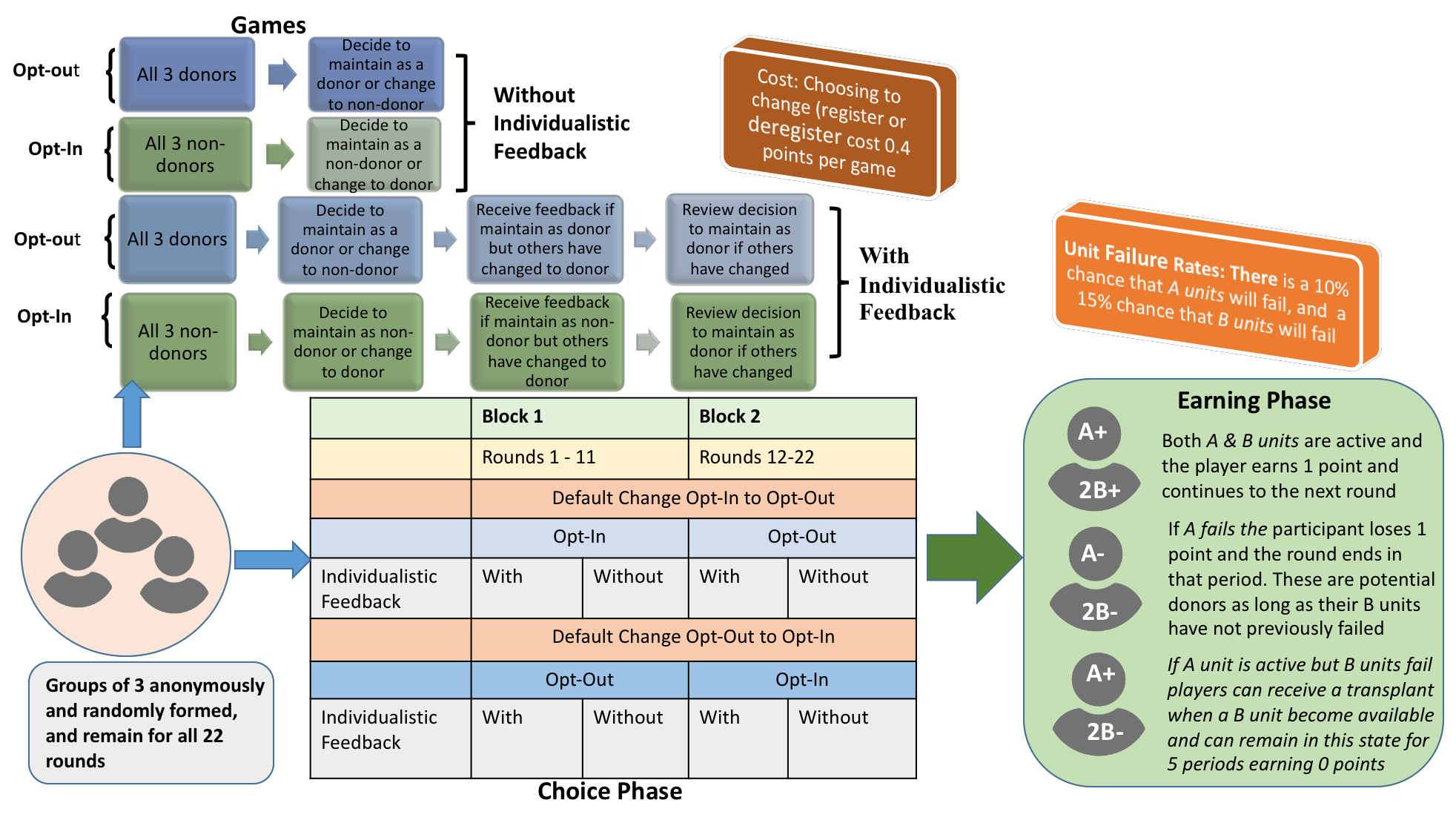


***General Instructions***. Participants read the instructions, which were also read aloud to them, and successfully completed a set of control questionnaire on the computer before doing the tasks to make sure that they understood the instructions. They were told that the rules of the task may change during the course of the experiment, and that they will be informed if – and when – the rules will change. Participants were not allowed to communicate in any way with other participants during the experiment. At the end of the experiment, participants completed a standard organ donation attitude measure and other post-game measures.

***Sessions***: Twelve lab-based sessions were run and in each session 18 participants were randomly allocated to 6 groups of 3 (apart from one session where there were 15 participants in 5 groups) and remained in these groups for the whole session. Group membership was anonymous with participants never knowing who their co-participants were.

***Choice Tasks and Earnings Stages***: Within each session, participants first completed all their *choice* *tasks*, after which they viewed their health outcomes in the *earnings* *stages*.

*Choice Tasks*: Participants first completed their choice tasks for 22 rounds, 11 rounds under an ‘opt-out’ policy in one block and 11 under ‘opt-in’ in another. The choice task in each round was for each participant to choose their *donor status*. Subjects were not informed how many rounds they had to play, and they were instructed on the new rules of the game, when moving from one policy system to the other. At the start of each round, everyone in a group was automatically a donor (opt-out policy) or a non-donor (opt-in policy) and participants were asked to make donor registration choice (to opt-in or opt-out depending on the policy they were exposed to). Being registered or remaining as a donor costs 0.4 points (see [D] below for payoff information). A screen showed the donor status and the *current donor count*, which is the number of other members of their group who had chosen to be a donor. Participants were asked to either maintain or change their donor status. Participants could change donor status only once per round. After completing all 22 choice tasks, subjects then viewed their earnings stages*.* The choices they made in the choice tasks went towards how much they could earn in the earnings stages (see [D] below).

*Earnings Stages*: Participants viewed each of the earnings stages for four *winning rounds* randomly chosen by the computer. In an earnings stage, each participant started with one active *A unit* (akin to a brain or heart), two active *B units* (akin to kidneys), and an *initial endowment* of 2 points (participants were informed that each point was worth 0.75p UK). The earnings stage proceeded in periods. In each period, there is a 10% chance that their *A unit* would fail, and a 15% chance that their *B units* would fail (both *B units fail* at the same time). If their *A unit* and *B units* are active in a period, they earn 1 point and the round continues to the next period. If their *A unit* fails, they lose 1 point and the round ends in that period. If their *A unit* is still active but *B units* fail, they can continue for up to 5 periods without *active B units* while waiting for a donor *B unit*. Before receiving a donor B unit, a participant will earn 0 points per period. They can receive a *donor B* unit from a co-participant who is a donor whose *A unit* has failed but *B units* have not failed before. If in one of these periods a participant receives a *B unit* from someone else, he or she will start earning points again. If one does not receive a *B unit* within those five periods, he or she will lose 1 point and their round ends in the last of those five periods.

**Instructions and Setting**

Participants read the instructions, which were also read aloud to them – and completed a set of control questionnaire before doing the tasks to make sure that they understood the instructions. They were told that the rules of the task may change during the course of the experiment, and that they will be informed if, and when, the rules will change. Participants were not allowed to communicate in any way with other participants during the experiment. At the end of the experiment participants completed a standard organ domain attitude measure and other post-game measures.

The game was programmed in z-tree (Fischbacher, 2007). At the end of each session all participants were paid based on the proceeds from the earnings stages.

**Design**

Our experiment was a 2 (*policy change*: opt-in to opt-out vs opt-out to opt-in) by 2 (*feedback*: with individualistic feedback versus without individualistic feedback) mixed design, with *poilicy change* as a within subject factor and *feedback* as a between subjects factor. Twelve lab-based sessions were run**,** organized into 6 sessions starting with opt-out and changing to opt-in and 6 sessions starting with opt-in and changing to opt-out. Of the 6 sessions starting with opt-out, 3 were with feedback and 3 were without feedback, and the same for the 6 starting with opt-in. All but one session had 6 independent groups each.

**Randomization**

Randomization was at the session level and within session between feedback and no-feedback

**Experimental** **Manipulations**

We manipulate policy and feedback as follows**.**

***Default Manipulation***: All participants received the following instructions

‘You will start each round with one active *A unit*, two active *B units*, and an *initial endowment* of 2 points. The task in each round is for you to choose your *donor status*, which is whether or not to register as a donor. Being registered as a donor will cost you 0.4 points. As a donor in the Earnings Stage, if your A unit fails, then your active B units will go to co-participants with failed B units and waiting for donor B units. Once a B unit has been donated, it cannot be donated again’

We then used the following framed instructions to manipulate opt-in versus opt-out policy. Under the *opt-in policy* participants were told that

‘At the start of each round, everyone in the group is automatically a non-donor.’,

whereas under the ‘*opt-out policy* participants were told that

‘At the start of each round, everyone in the group is automatically a donor.’

**Feedback Manipulation:** The 50% of the participants receiving *‘individualistic feedback’* were provided with feedback on how many of the other members of their group had chosen to remain as a donor (under opt-out) and become a donor (under opt-in) during each round. Based on that feedback participants could change their registration decision until everyone decided that they were happy to remain with their registration decision. The 50% received *‘no individualistic feedback’* during each round but only knew the others’ decisions at the end of each choice task.

**Measures**

***Organ Donor Attitudes***. We index organ donor attitudes using the organ donor attitude scale developed by Morgan et al (2008) to assess (1) **Jinx factor (**Higher scores suggest a greater feeling that it is bad luck to talk about death or becoming an organ donor (α = .55 (2) **Ick factor:**. Higher scores indicate greater feelings of disgust at the idea of organ donation (α = .87) (3) **Medical mistrust:** Higher scores indicate greater feelings of medical mistrust (α = .77) (4) **Bodily integrity:**  Higher scores indicate a greater belief in the need to maintain bodily integrity (α = .79) and **Perceived benefit:** Higher scores indicate a greater perceived benefit of being an organ donor (α = .45). As two sub-factor ‘jinx’ and ‘perceived benefits’ have low coefficient alphas (below .70) we did not include these scales as covariates.

***Current Organ Donor Status***. We asked participants to indicate YES or NO if they were currently registered as an organ donor.

***Validity check*.** To check that participant interpreted the abstract nature of the game as representing organ donation we asked them to indicate which type of real-world donation the game was similar to: (1) organ donation, (2) blood donation, (3) donating money to charity, (4) giving up time to volunteer for a charity. They had to choose only one of these.

***Outcomes*:** Our main outcome variable was the per-round registration choices made by each participant. That is being registered by either opting in or not opting out.

**[B] Instructions Given to Participants**

Participant received the following instructions. The different manipulations are distinguished below by [opt-out], {opt-in}, |no interim-feedback|, and <interim-feedback>, but participants only saw the text relevant to their tasks.

**General Instructions:**

Welcome and thank you for participating! This is an experiment on decision making. It is organized by Nottingham University’s Schools of Economics (NSE), Psychology (NSP) and Business (NUBS), funded by NSP and NUBS, and approved by the NSP Ethics Committee. The whole experiment will last approximately 90 minutes.

Participation in this experiment is totally voluntary and you are under no obligation to take part. You are free to withdraw at any point before or during the study. All data collected will be kept confidential and used for research purposes only. If you have any questions or concerns, please do not hesitate to ask now or at any point of time.

The experiment has a number of rounds. In each round, you will perform some *tasks* on the computer. You will be able to earn some *points*. Each point is worth £0.75. You will be paid based on your earnings in four *winning rounds* randomly chosen by the computer. You will be told which four rounds after completing all your tasks.

At the end of the experiment, please fill in some forms and a questionnaire while the experimental supervisor prepares your payment. Please do not leave until you have received your payment and returned the completed receipt, forms and questionnaire.

Please read the instructions carefully and complete the questionnaire before doing the tasks. The purpose of the questionnaire is to make sure that you have understood the instructions. The rules of the task may change during your course of the experiment, and you will be informed if and when they do.

Please do not communicate in any way with other participants during the experiment. Do not use your mobile phone or any other software apart from the experimental software on the computer. To maintain the scientific integrity of the experiment, it is important that you follow these rules. We will ask you to leave without payment if you break the rules.

**Presented just before round 1**

**Your Tasks:**

The computer will randomly assign you into groups of 3. The co-participants in your group will stay the same throughout the experiment. You will not know who your co-participants are. In each round your task is to make choices that go towards determining how much you earn in the *Earnings Stage* of that round.

You will start each round with one active *A unit*, two active *B units*, and an *initial endowment* of 2 points. The task in each round is for you to choose your *donor status*, which is whether or not to register as a donor. Being registered as a donor will cost you 0.4 points. As a donor in the Earnings Stage, if your A unit fails, then your active B units will go to co-participants with failed B units and waiting for donor B units. Once a B unit has been donated, it cannot be donated again.

At the start of each round, everyone in the group is automatically a {non-}donor. The screen shows your donor status and the *current donor count*, which is the number of donors in the group at present.

<A task proceeds in periods.> You can MAINTAIN or CHANGE your donor status by clicking on the respective buttons. <However, you can CHANGE your donor status only once per task. The task ends if everyone’s donor status is left unchanged in the same period. Otherwise, the task continues to the next period where you will see the new *current donor count*.> |A task ends when everyone has chosen.| When a task ends, you will see the *final donor count* before moving to the task for the next round.

**Your Earnings:**

Each Earnings Stage proceeds in periods. In each period, there is a 10% chance that your A unit will fail, and a 15% chance that your B units will fail (both B units fail at the same time). If your A unit and B units are active in a period, you will earn 1 point and your round continues to the next period. If your A unit fails, you will lose 1 point and your round ends in that period.

If your A unit is still active but your B units fail, you can continue for up to 5 periods without active B units while you wait for a donor B unit. Before receiving a donor B unit, you will earn 0 points. You can receive a donor B unit from a co-participant who is a donor whose A unit has failed but B units have not failed before. If in one of these periods you receive a B unit from someone else, you will start earning points again. As a reminder, once a B unit has been donated, it cannot be donated again. If you do not receive a B unit within those five periods, you will lose 1 point and your round ends in the last of those five periods.

You will view the outcomes of each Earnings Stage of your four winning rounds after completing all the choice tasks in the experiment.

*Please raise your hand if you have any questions.*

**Presented just before round 12**

**Change of rules:**

From now on, at the start of each round, everyone in the group is automatically a [non-] donor.

**Control Questionnaires**

*Control questionnaire presented before starting tasks:*

1) Please enter the correct numbers.

a) How much does it cost to be a donor? ___ points

b) How much will you earn for 5 periods with an active A unit and two active B units? ___ points

c) How much will you lose for the period in which you have an A unit failure? ___ points

Ans: 0.4/5/1

2) Which of the following statements are true?

a) Being on the donor list means you can donate your B units to people who might need them, in the event that you have A unit failure.

b) Being on the donor list means you can donate your A unit to people who might need them, in the event that you have B unit failure.

c) Not being on the donor list means you cannot receive B units if you need them, from people who have A unit failure.

Ans: a

3) How many randomly selected rounds will you be actually paid for?

Ans: 4

4) Which of the following statements are true?

a) At the start of each round, everyone is automatically a donor.

b) If I want to be a donor, I have to choose [MAINTAIN] {CHANGE}.

<c) After changing my donor status to [donor] {non-donor}, I will not be able to change my donor status back to [non-donor] {donor} again in the same round.>

Ans: [a],b,c

5) Under which of the following conditions will the Choice Stage end?

a) Some participants choose CHANGE and others choose MAINTAIN <in the same period>.

b) All participants choose MAINTAIN <in the same period>.

c) |All participants choose CHANGE.| <All remaining participants who have not chosen CHANGE before choose MAINTAIN again in the same period.>

Ans: |a|,b,c

*Control questionnaire for change of rules:*

Which of the following statements are true?

a) At the start of each round, everyone is automatically a donor.

b) If I want to be a donor, I have to choose [CHANGE] {MAINTAIN}.

<c) After choosing CHANGE, I will be able to choose to revert to being a [non-]donor in the same round.>

Ans: {a},b

**[C] Game Screen Shots**

This file contains the screen seen by the participants during the game

1. Control Questions


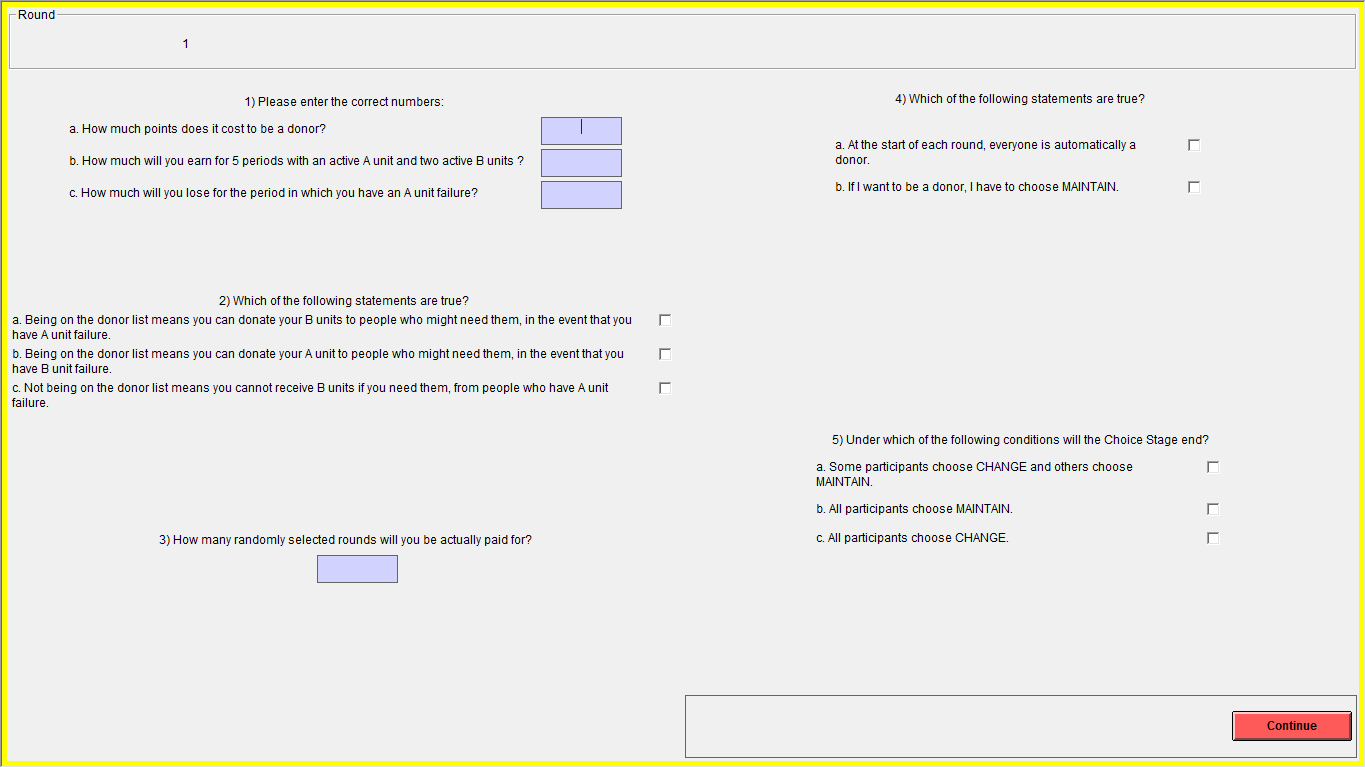


1. Decision screen – opt-out


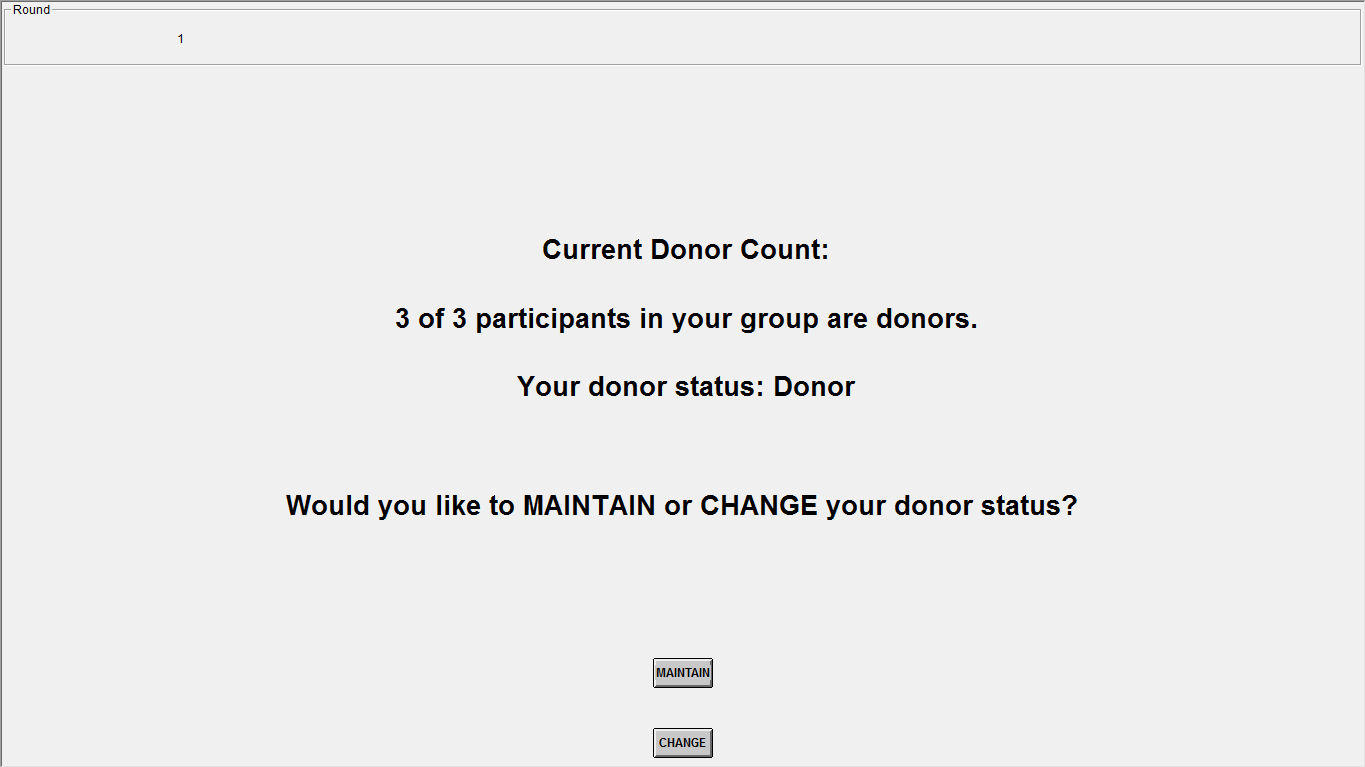


1. End round feedback


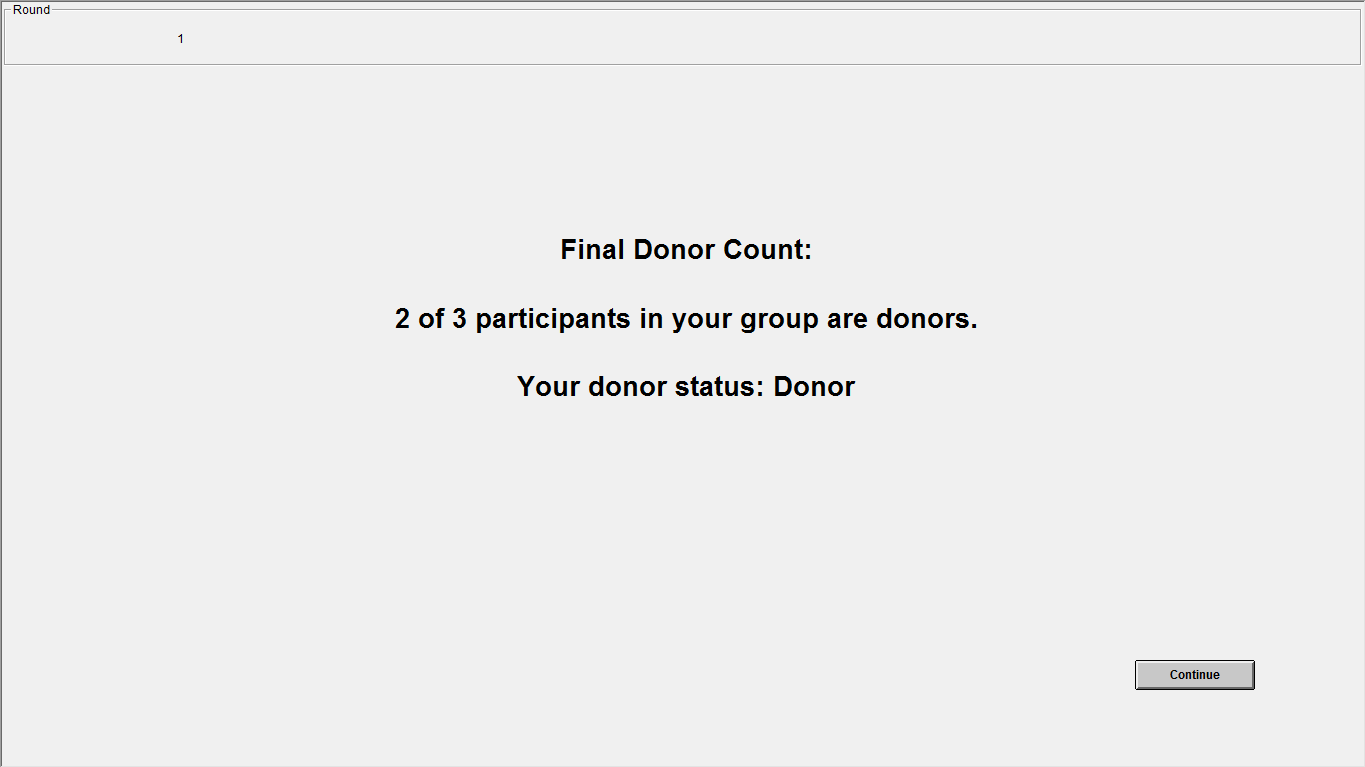


1. Transition to opt-in


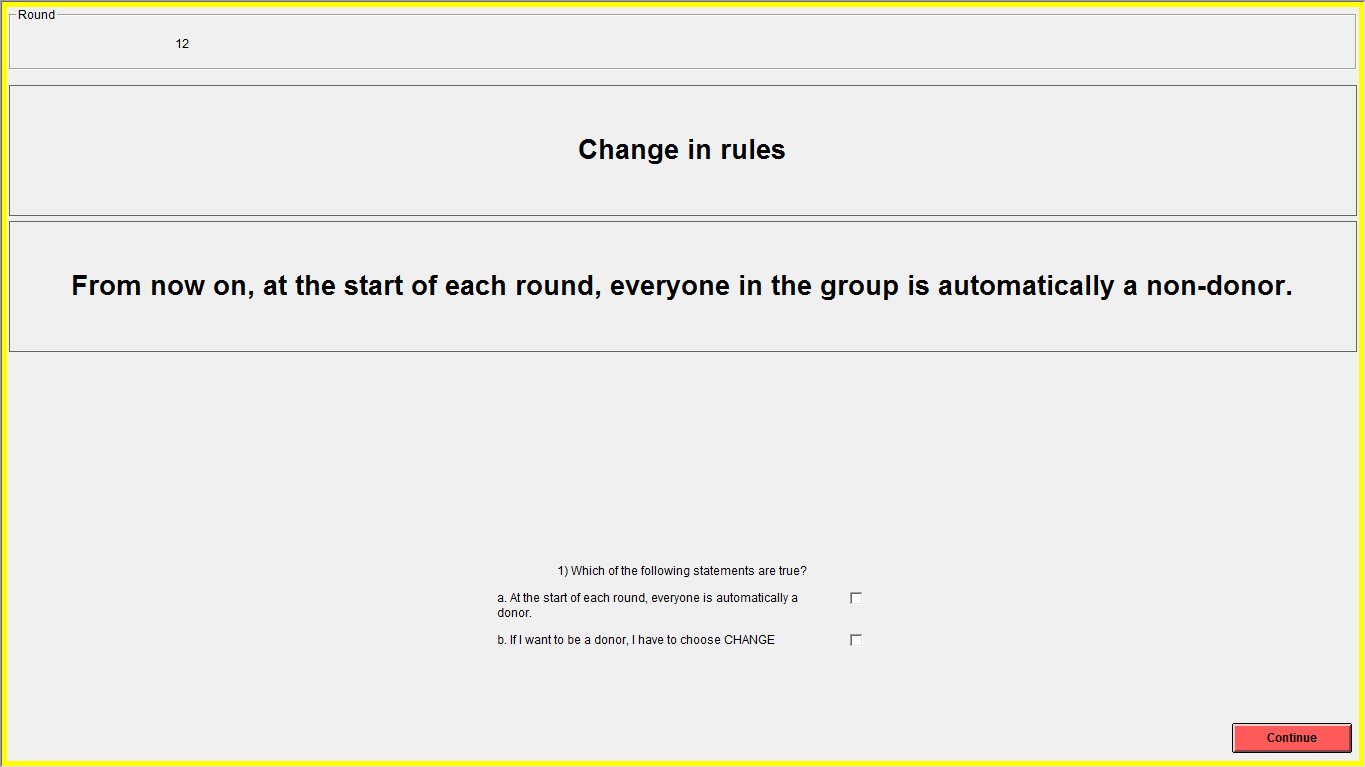


1. Decision screen - opt-in


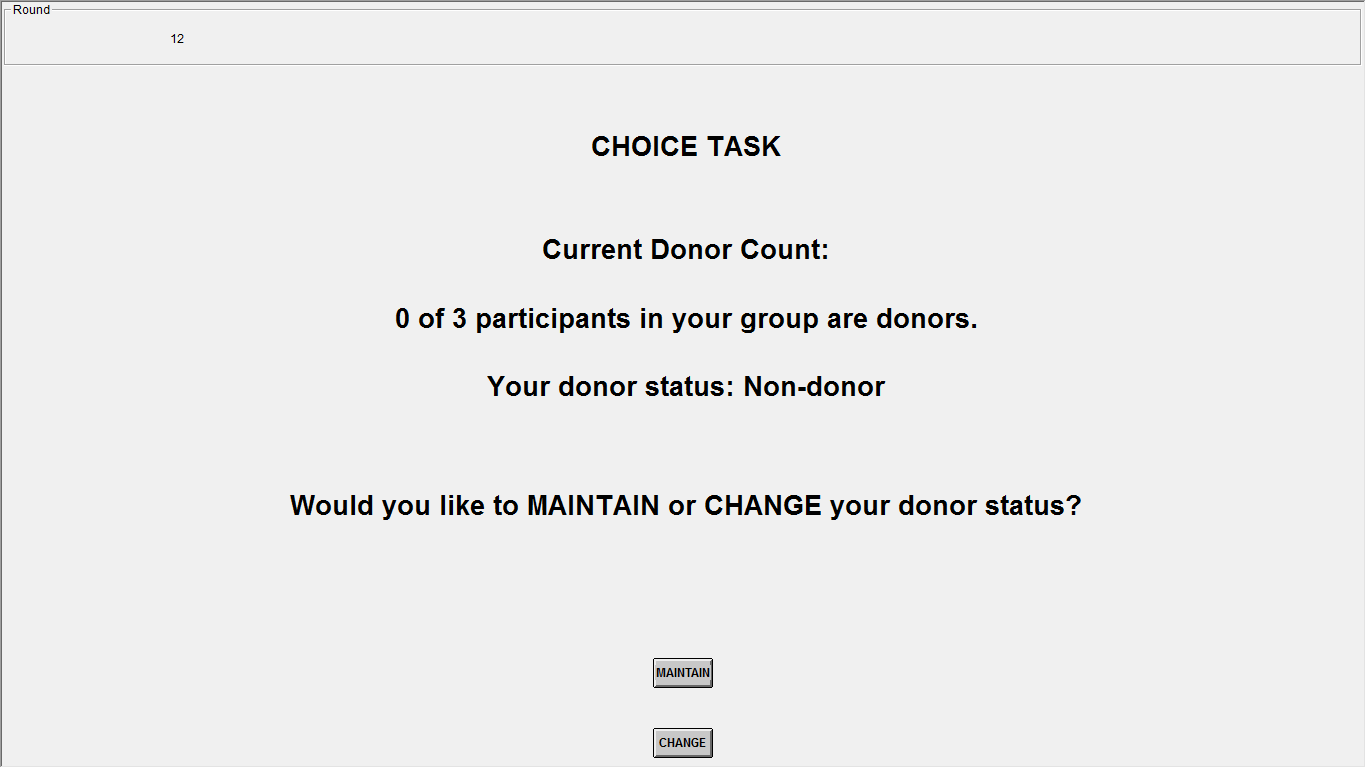


1. Rounds chosen for payment


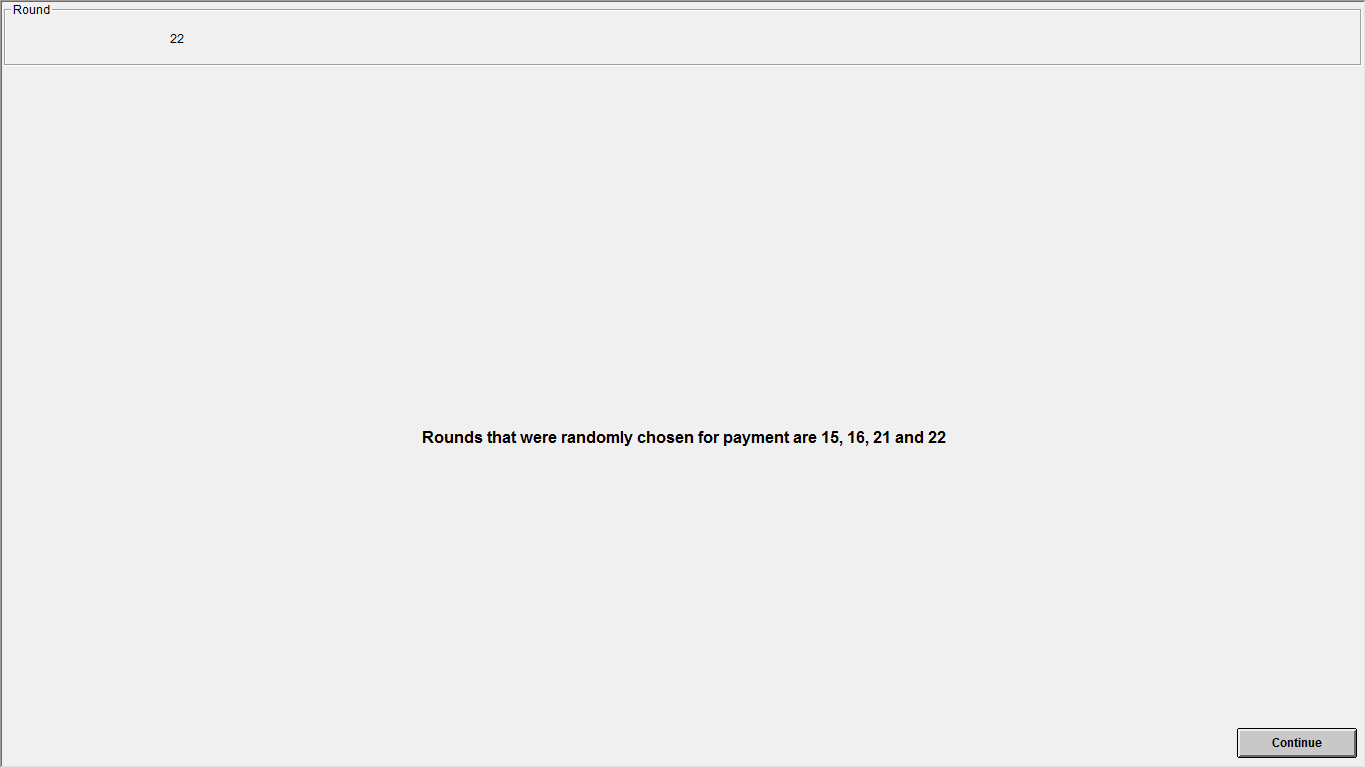


1. Payment round number


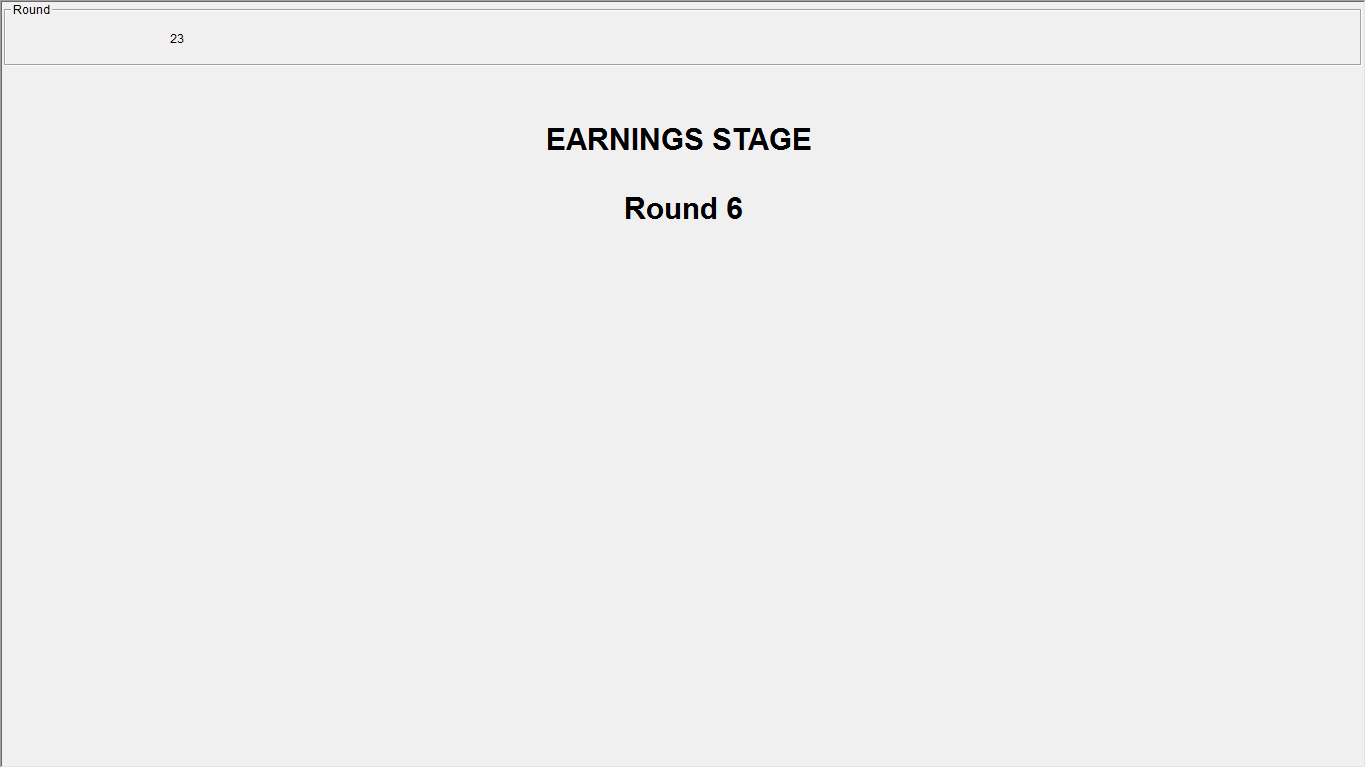


1. Active brain and kidney


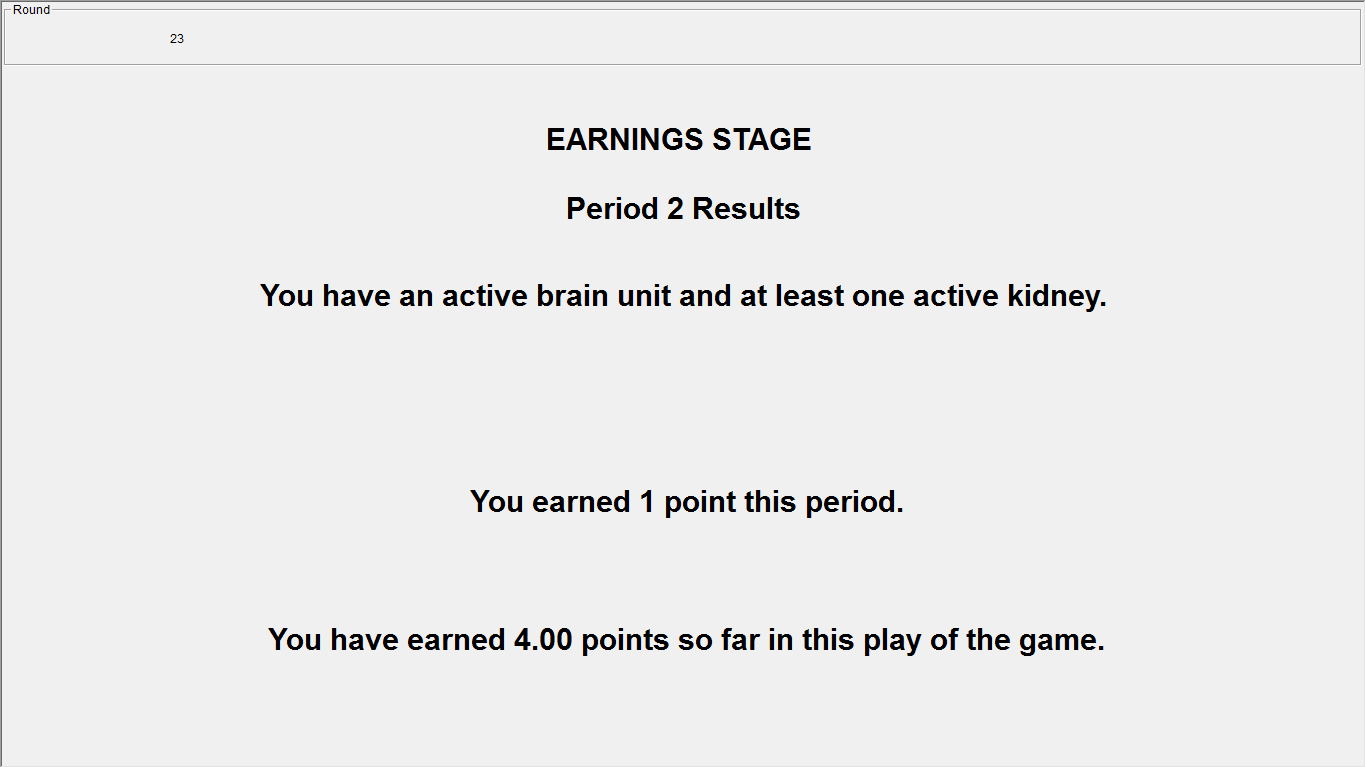


1. Kidney failure


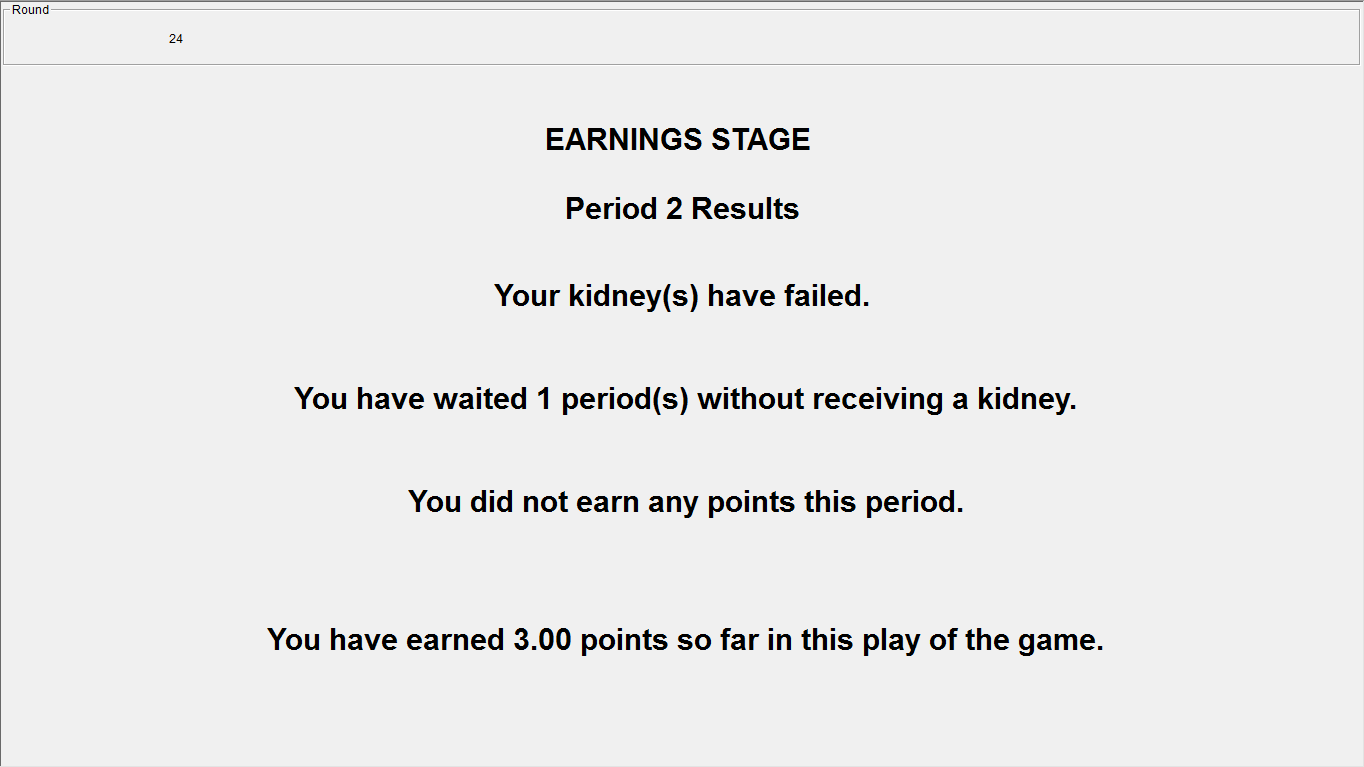


1. Waiting for donation of a kidney


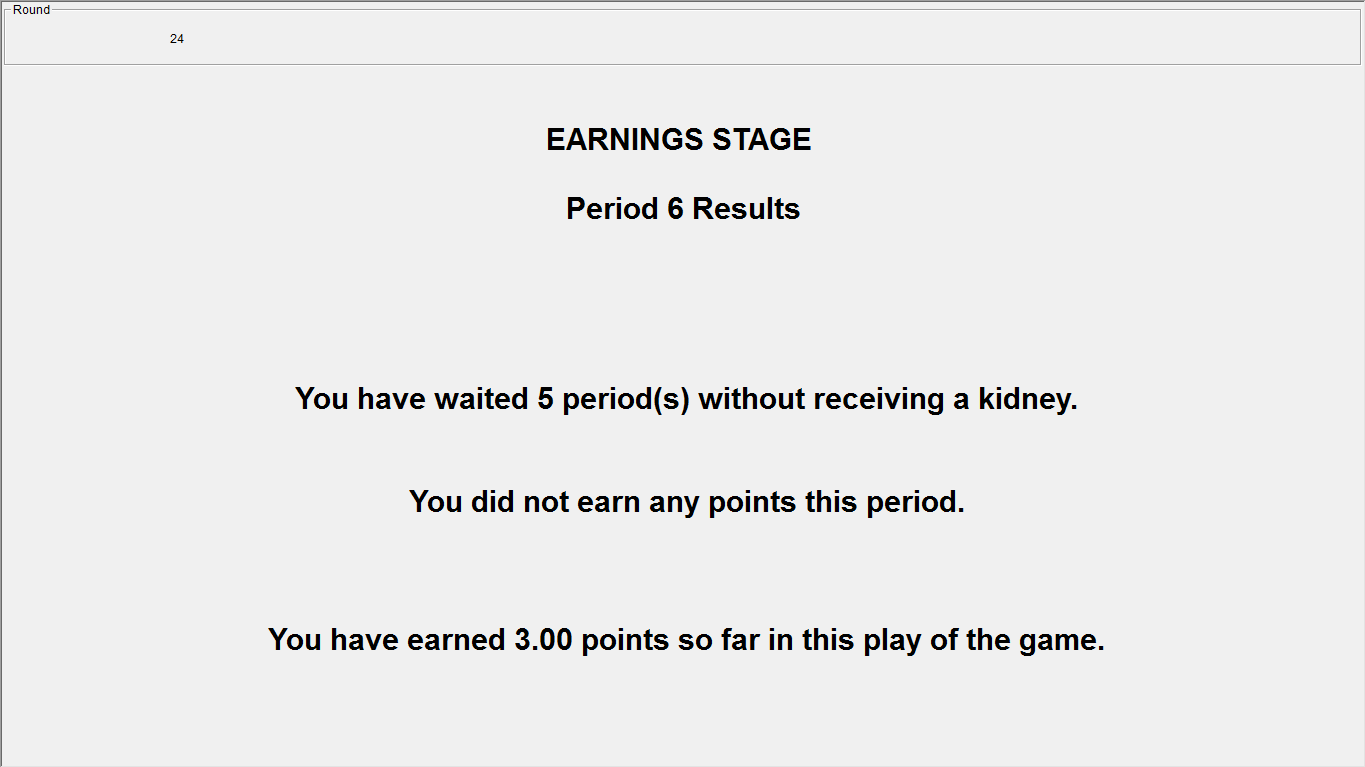


1. Too many periods without a kidney


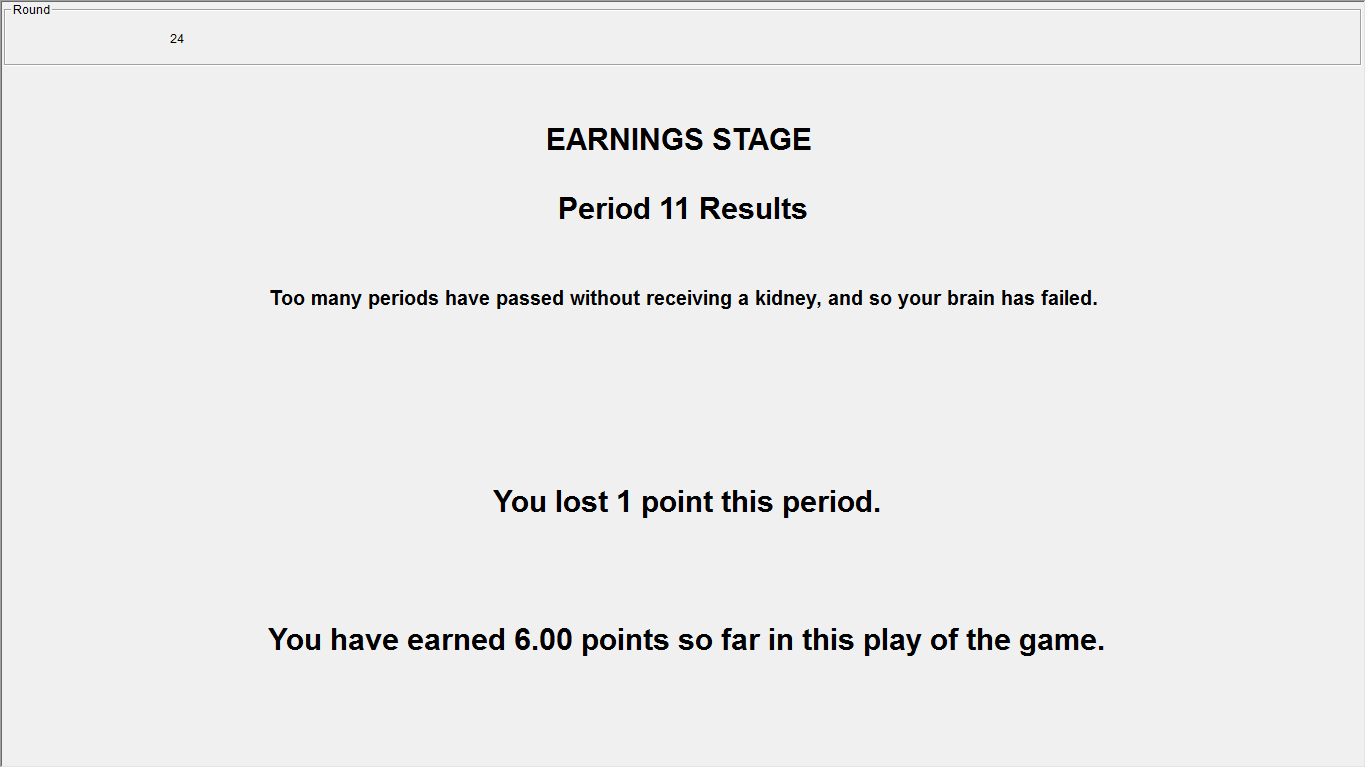


1. Kidney received


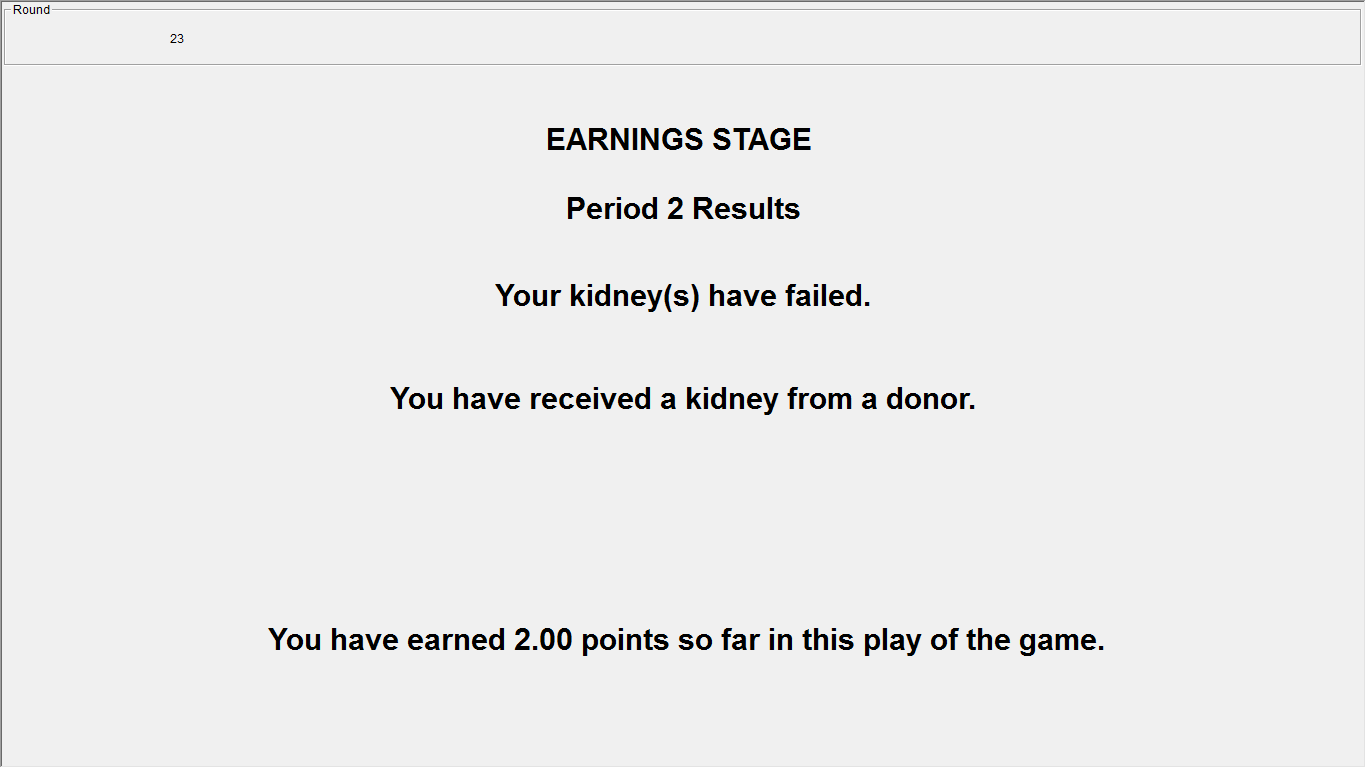


1. Kidney given


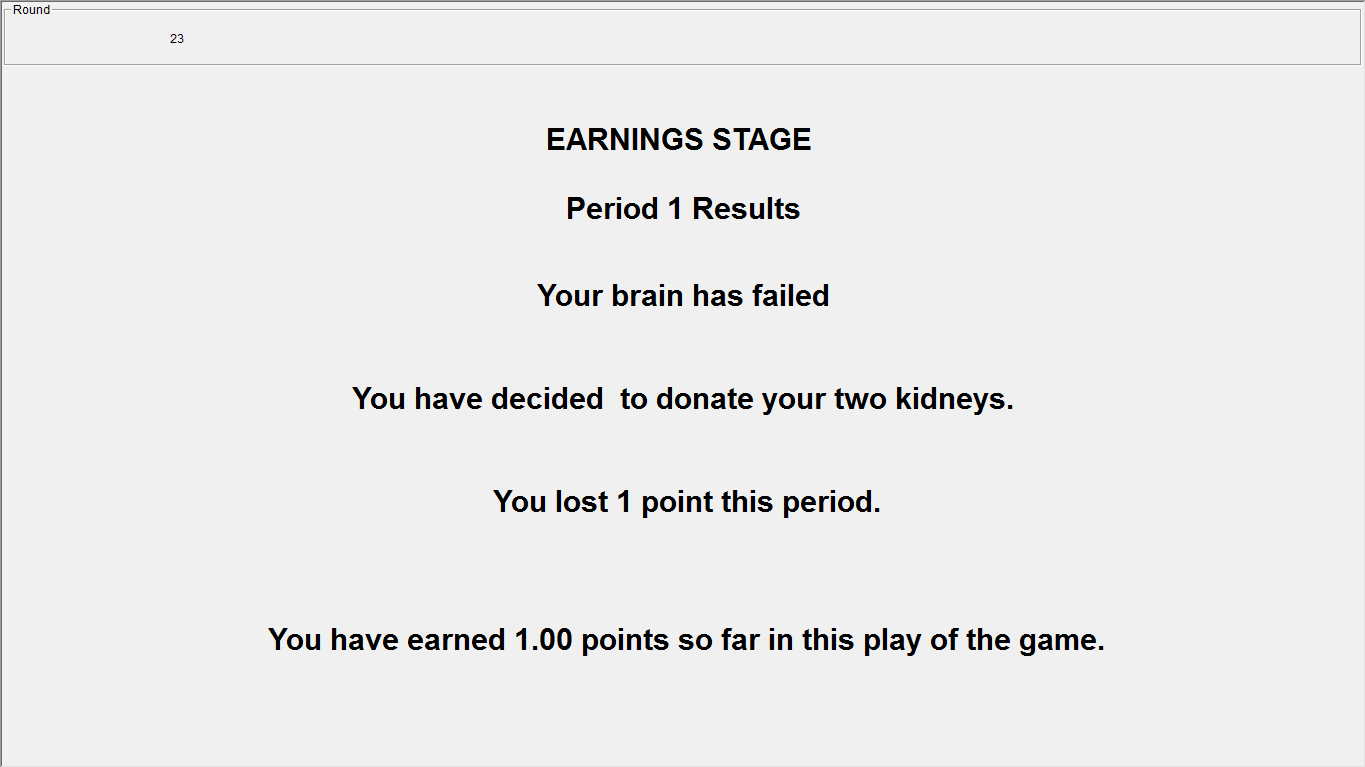


1. Brain failure


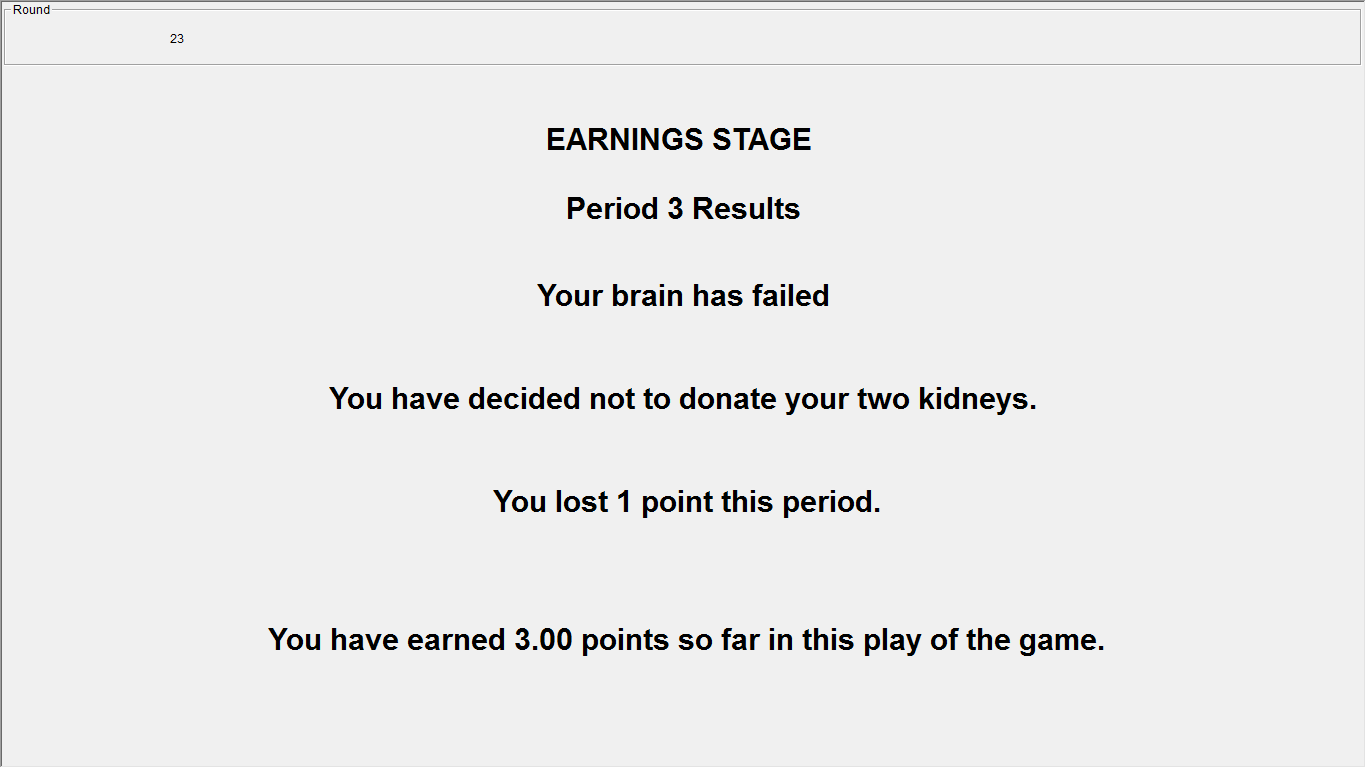


1. Individualistic feedback


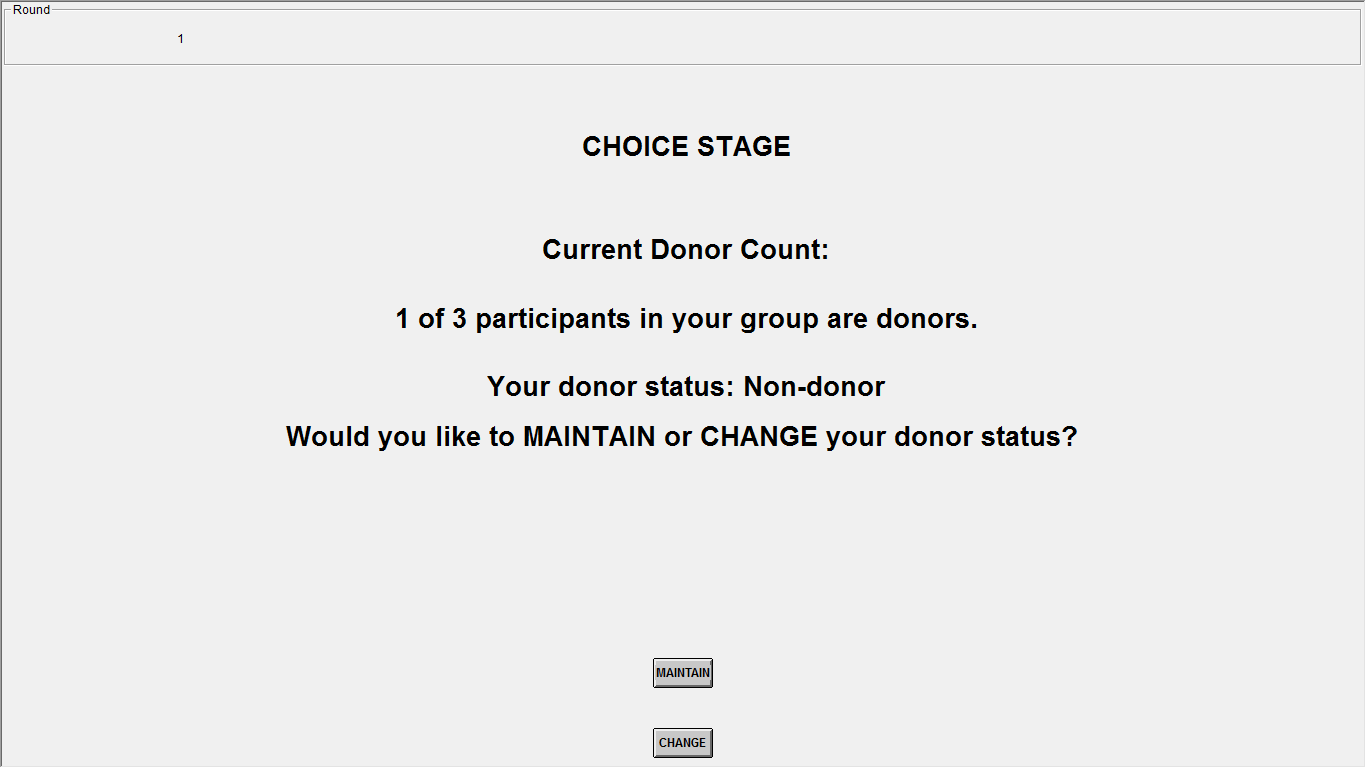


**[D] Earnings Stage Display Error.**

Due to a programming error, the earnings stage screen displayed the terms ‘brain’ instead of ‘A unit’ and ‘kidneys’ instead of ‘B units’. This has zero impact on our main outcome measure of registration rates for the following reasons.

- It occurred after all choice stage decisions were completed (our main outcome data).

As shown in the results our data reproduces the two main epidemiological findings in organ registration work: (i) the beneficial effect of the opt-out policy, compared to opt-in, in the absence of feedback and (ii) the beneficial effect of registrations with status update feedback in an opt-in system – the ‘Facebook effect’. As such, while regrettable, the impact on our results is zero

**[E] Post-game Questions**

This file details the question asked after the game.

**POST-STUDY QUESTIONS**

**ID:________________________________**

P**lease circle one option, from the list below, the type of real-world donation do you think this game is most similar to?**

| Organ donation, |
| --- |
| Blood Donation, |
| Donating Money to Charity, |
| Giving up time to volunteer for a charity |

**About you**

| Have you ever donated blood? | YES NO (please circle one) |
| --- | --- |
| Are you currently a blood donor? | YES NO (please circle one) |
| Have you donated blood in the last Year? | YES NO (please circle one) |
| Are you a registered organ donor? | YES NO (please circle one) |
| Have you ever volunteered to help a charity? | YES NO (please circle one) |
| Have you ever donated money to a charity? | YES NO (please circle one) |
| What is your sex? | Male female (please circle one) |
| How old are you?_______ years |  |

**[F]** **Power Calculations**

To assess group based policy implications power was based on Tan et al. (2015) for a two-sample comparison of means with a power of .80 and an alpha of .05. This indicated we required 18 groups per condition, with 3 per group required a final N of 216.

**[F] Experimental Payments**

Each experimental point was worth £0.75 (UK). Participants were incentive compatibly paid for the outcomes of four randomly chosen rounds known to them only after all choices had been made, i.e. realized in the four respective earnings stages (as described in the experimental instructions) after round 22. This ‘random lottery incentive’ design helps to prevent wealth effects of previous earnings influencing choices in later rounds, by inducing subjects to think of each task independently. Our design and game set up are, therefore, specified to be faithful to organ donor registrations in the real world where organ registration choices are *ex ante* not influenced by experiences of organ failure and donating or receiving an organ. An earnings stage involves a simulated lifecycle wherein a participant experiences organ failure and donation outcomes. For example, as a donor in the earnings stage, if their A unit fails, then their active B units will go to co-participants with failed B units and waiting for donor B units. Once a B unit has been donated, it cannot be donated again. Due to a programming error, the earnings stage screen displayed the terms ‘brain’ instead of ‘A unit’ and ‘kidneys’ instead of ‘B units’. However, this has zero impact on our main results.

**Supplementary File S4: Additional Supporting Analyses for Organ Donation Game Experiment**

This file contains the detailed results to support the conclusion presented in the main paper.

Details on the descriptive statistics on mean registration rates for the 22 rounds as a function of policy change (opt-in to opt-out and visa-versa) and feedback (without versus with individualistic feedback) can be found in Table S1. The move to opt-out from opt-in results in a steep decline in registration rates, especially with individualistic feedback.

|  | Without individualistic feedback  Out to in | Without individualistic feedback  In to out | With individualistic feedback  Out to in | With individualistic feedback  In to out |
| --- | --- | --- | --- | --- |
| Block 1: Games 1-11  *SE*  95% CI | **.5639731**  *.0203638*  .5240505  .6038957 | **.4242424**  *.0202955*  .3844537  .4640311 | **.5084175**  *.0205296*  .4681698  .5486652 | **.4955437**  *.021128*  .4541228  .5369645 |
| Block 2: Games 12-22  *SE*  95% CI | **.46633**  *.0204859*  .4261679  .506492 | **.4292929**  *.0203262* .389444  .4691418 | **.5673401**  *.0203455*  .5274534  .6072268 | **.3761141**  *.02047*  .3359832  .416245 |

**Table S1. Registration rates across treatments.** Out to in = changing from an opt-out to an opt-in policy and in to out = changing from an opt-in to an out-in policy and

Table S2 presents the random effects logit models (with group level clustered random errors) for default policy (change) and feedback effects that underlie the results reported in Figures 2 and 3 in the main text. To test our hypothesis that a change from an opt-in to opt-out default policy results in reduced registration rates with individualistic feedback, we model the difference in registration rates across policy changes as a function of feedback. We operationalize this as the difference between mean individual registration rates across blocks (a negative score indicates a decrease in donor rates and positive score an increase). Treatment dummies for moving from opt-out to opt-in without individualistic feedback, move from opt-in to opt-out without individualistic feedback, moving from opt-out to opt-in with individualistic feedback and moving from opt-in to opt-out with individualistic feedback were specified. The dummy for moving from opt-in to opt-out without individualistic feedback was used as the base for comparison as the mean donor rates are equivalent at approximately 42% (no slope).

The results are shown in Table S2. To ensure that any effects are not due to either including or excluding covariates, we ran models with and without covariates. The results show that moving from opt-in to opt-out, with individualistic feedback, results in reduced registration rates. The significant drop corresponds to a move from Bar F to Bar E in Figure 1 (main text).

Under opt-in, we expect a positive ‘good shepherd’ effect off equilibrium that is compatible the Facebook effect. In contrast, without feedback, the same effect is absent, which we termed the ‘opt-in disadvantage’ as opposed to the standard opt-out advantage. Moving to opt-in where cooperation is subject to these effects from opt-out, where there is a negative ‘lone wolf’ effect with feedback versus a positive default effect (reproducing the epidemiological result) without feedback, implies an increase in the case of feedback but a decrease in the case without feedback.

|  |  |  | ***95% CI*** | |  | |  | ***95% CI*** |  |
| --- | --- | --- | --- | --- | --- | --- | --- | --- | --- |
| ***Variable*** | ***Coefficient (SE_robust_)*** | ***p*** | ***Lower*** | ***Upper*** | ***Coefficient (SE_robust_)*** | | ***p*** | ***Lower*** | ***Upper*** |
| *Out-to-In (without individualistic feedback)* | **-0.103 (0.045)** | **0.024** | **-0.192** | **-0.014** | **-0.178 (0.061)** | **0.004** | | **-0.298** | **-0.058** |
| *Out-to-In (with individualistic feedback)* | 0.054 (0.054) | 0.323 | -0.053 | 0.161 | 0.022 (0.59) | 0.709 | | -0.093 | 0.137 |
| *In-to-Out (with individualistic feedback)* | **-0.124 (0.062)** | **0.043** | **-0.245** | **-0.004** | **-0.131 (0.067)** | **0.050** | | **-0.263** | **-0.000** |
| *Age* |  |  |  |  | 0.025 (0.013) | 0.052 | | -0.000 | 0.051 |
| *Sex* |  |  |  |  | 0.077 (0.081) | 0.343 | | -0.082 | 0.327 |
| *Game Beliefs* |  |  |  |  | 0.117 (0.127) | 0.359 | | -0.133 | 0.366 |
| *Organ Donor Register* |  |  |  |  | 0.086 (0.093) | 0.358 | | -0.097 | 0.269 |
| *Constant* | 0.005 (0.037) | 0.893 | -0.068 | 0.078 | -0.683 (0.310) | 0.027 | | -1.290 | -0.076 |
| *Overall R^2^* | .109 |  |  |  | .199 |  | |  |  |
| *Number of Observations (Groups)* | 213 (71) |  |  |  | 213 (71) |  | |  |  |

**Table S2**. *Random Effects* *GLS Regression* *with Group Level Clustered Random Errors* *of Registration Changes from Block 1 to Block 2*. The dependent variable is the change in registration rates from blocks 1 and 2 = mean registration in block 2 – mean registration in block 1. The predictors are treatment dummies for moving from opt-out to opt-in without individualistic feedback, moving from opt-out to opt-in with individualistic feedback and moving from opt-in to opt-out with individualistic feedback. The base for comparison is the treatment where we move from opt-in to opt-out without individualistic feedback, where the mean donor rates are equivalent at 42% (no slope). Sex: Female = 0, Male = 1. Game beliefs: Not believe it is a game about organ donation = 0, Believe it is a game about organ donation = 1. Organ Donor Register: state not on the register = 0, state on the register = 1. Group-level random effects.

**Opt-Out**

**Opt-In**

**Opt-In**

**Opt-Out**

**Figure S1**. *Effects of Policy Change as a Function of Status Updating Feedback for the 22 Rounds* ***(****mean registration rates per round)*

**Models 1 to 5 in the main text without covariates**

Tables S3, S4 and S5 presents the results that correspond to Tables 1, 2 and 3 respectively in the main paper, but analyse these data minus the covariates. The results are identical to those reported in Tables 1, 2 and 3 in the main text.

|  |  |  | ***95%*** |  |  |  | ***95%*** |  |
| --- | --- | --- | --- | --- | --- | --- | --- | --- |
|  | ***Coefficient (SE_robust_)*** | ***p*** | ***Lower*** | ***Upper*** | ***Coefficient (SE_robust_)*** | ***p*** | ***Lower*** | ***Upper*** |
| *Decision to Register* | Model 1 |  |  |  | Model 2 |  |  |  |
| *Round* | **-0.004 (0.002)** | **0.043** | **-0.008** | **-0.000** | -0.002 (0.001) | 0.212 | -0.004 | 0.001 |
| *Feedback* | -0.053 (0.063) | 0.402 | -0.178 | 0.071 | -0.031 (0.036) | 0.401 | -0.102 | 0.041 |
| *Opt-In* | **-0.051 (0.023)** | **0.023** | **-0.096** | **-0.007** | -0.019 (0.013) | 0.140 | -0.045 | 0.006 |
| *Lag Round Decisions* |  |  |  |  | **0.439 (0.061)** | **0.000** | **0.318** | **0.559** |
| *Feedback * Opt-In* | **0.141 (0.039)** | **0.000** | **0.066** | **0.217** | **0.083 (0.026)** | **0.002** | **0.032** | **0.134** |
| *Constant* | 0.546 (0.049) | 0.000 | 0.450 | 0.642 | 0.289 (0.044) | 0.000 | 0.202 | 0.375 |
| *R^2^ _overall_* | .016 |  |  |  | .207 |  |  |  |
| *N of Observations (Groups)* | 3,102 (71) |  |  |  | 2,961 (71) |  |  |  |

**Table S3:** *Overall (Model 1) and* *Conditional Cooperation (Model 2) Effects (Random Effects GLS Models with group level clustered random error) on group averages of decisions to register plus Covariates.* The dependent variable is the group average of the *Decision to Register* (for each individual, 1 = on the register: actively registered under opt-in and not opted-out under opt-out, or 0 = not on the register: not having registered under opt-in and opted-out under opt-out). We have the following independent variables. *Round* is the game number 1-22. *Feedback*: 0 = without individualistic feedback and 1 = with individualistic feedback, *Opt-In:* 0 = Opt-out and = 1 = Opt-in. *Lag Round Decisions =* percent of group registered in the previous round.

|  | ***Without Individualistic Feedback*** | | | | ***With Individualistic Feedback*** | | | |
| --- | --- | --- | --- | --- | --- | --- | --- | --- |
|  |  |  | ***95%*** |  |  |  | ***95%*** |  |
|  | ***Coefficient (SE_robust_)*** | ***p*** | ***Lower*** | ***Upper*** | ***Coefficient (SE_robust_)*** | ***p*** | ***Lower*** | ***Upper*** |
| *Decision to Register* | Model 3 |  |  |  | Model 4 |  |  |  |
| *Round* | -0.001 (0.001) | 0.357 | -0.003 | 0.001 | -0.002 (0.002) | 0.300 | -0.006 | 0.002 |
| *Opt-In* | **0.053 (0.024)** | **0.029** | **0.005** | **0.101** | 0.013 (0.041) | 0.761 | -0.068 | 0.093 |
| *Lag Round Decisions* | **0.700 (0.061)** | **0.000** | **0.581** | **0.820** | **0.336 (0.086)** | **0.000** | **0.168** | **0.503** |
| *Lag Round Decisions * Opt-In* | **-0.130 (0.050)** | **0.010** | **-0.229** | **-0.031** | 0.113 (0.085) | 0.188 | -0.055 | 0.281 |
| *Constant* | **0.150 (0.038)** | **0.000** | **0.076** | **0.223** | **-0.307 (0.056)** | **0.000** | **0.197** | **0.418** |
| *R^2^ _overall_* | .401 |  |  |  | .173 |  |  |  |
| *N of Observations(Groups)* | 756 (36) |  |  |  | 2,205 (35) |  |  |  |

**Table S4:** *Across Round Conditional Cooperation Effects as a function of Default and Feedback (Random Effects GLS Models with group level clustered random error) on group averages of decisions to register plus Covariates - the across round Lone-Wolf Effect.* The dependent variable is the group average of the *Decision to Register* (for each individual, 1 = on the register: actively registered under opt-in and not opted-out under opt-out, or 0 = not on the register: not having registered under opt-in and opted-out under opt-out). We have the following independent variables. *Round* is the game number 1-22, *Opt-In:* 0 = Opt-out and = 1 = Opt-in. *Lag Round Decisions =* percent of group registered in the previous round.

|  | ***With Individualistic Feedback*** | | | |
| --- | --- | --- | --- | --- |
|  |  |  | ***95%*** |  |
|  | ***Coefficient (SE_robust_)*** | ***p*** | ***Lower*** | ***Upper*** |
| *Decision to Register* | Model 5 |  |  |  |
| *Round* | -0.002 (0.002) | 0.226 | -0.006 | 0.001 |
| *Opt-In* | **0.428 (0.056)** | **0.000** | **0.318** | **0.538** |
| *Lag Period Decisions* | **0.603 (0.060)** | **0.000** | **0.486** | **0.720** |
| *Lag Period Decisions * Opt-In* | **-0.180 (0.085)** | **0.034** | **-0.347** | **-0.013** |
| *Constant* | 0.180 (0.040) | 0.654 | -0.060 | 0.096 |
| *R^2^ _overall_* | .241 |  |  |  |
| *N of Observations(Groups)* | 1,350 (35) |  |  |  |

**Table S5:** *Conditional Cooperation Within Rounds (Random Effects GLS Models with group level clustered random error) on group averages of decisions to register plus Covariates - the within round Lone-Wolf Effect.* The dependent variable is the group average of the *Decision to Register* (for each individual, 1 = on the register: actively registered under opt-in and not opted-out under opt-out, or 0 = not on the register: not having registered under opt-in and opted-out under opt-out). We have the following independent variables. *Round* is the game number 1-22, *Opt-In:* 0 = Opt-out and = 1 = Opt-in, *Lag Period Decisions* = percent of group registered in the previous period played within the present round.

**Modelling Individual Responses**

These analyses reported the model from the main text based in individual responses rather than within game averages. We estimate across round conditional cooperation effects with others’ decisions in previous rounds (*Lag Others’ Decisions*) and within round conditional cooperation effects with others’ decisions in the present round (*Others’ Decisions*). Tables S6 to S8 report Models 1 to 5 with covariates and Tables S9-S11 reports them without covariates. These analyses replicate the main within round ‘lone wolf’ effects, however, the p-values of conditional cooperation effects across rounds weaken when estimated with individual decisions (Table S7) compared to the groups level estimates (Table S4).

**Modelling Individual Responses (Models 1 to 5 with covariates)**

|  |  |  | ***95%*** |  |  |  | ***95%*** |  |
| --- | --- | --- | --- | --- | --- | --- | --- | --- |
|  | ***Coefficient (SE_robust_)*** | ***p*** | ***Lower*** | ***Upper*** | ***Coefficient (SE_robust_)*** | ***p*** | ***Lower*** | ***Upper*** |
| *Final Decision to Register* | Model 1 |  |  |  | Model 2 |  |  |  |
| *Round* | **-0.026 (0.009)** | **0.006** | **-0.045** | **-0.008** | **-0.024 (0.010)** | **0.012** | **-0.043** | **-0.005** |
| *Feedback* | -0.431 (0.383) | 0.260 | -1.182 | 0.320 | -0.433 (0.396) | 0.274 | -1.210 | 0.343 |
| *Opt-In* | **-0.289 (0.124)** | **0.019** | **-0.532** | **-0.047** | **-0.273 (0.115)** | **0.018** | **-0.498** | **-0.047** |
| *Others’ Decisions* |  |  |  |  | -0.042 (0.087) | 0.630 | -0.214 | 0.129 |
| *Feedback * Opt-In* | **0.783 (0.212)** | **0.000** | **0.366** | **1.199** | **0.826 (0.206)** | **0.000** | **0.423** | **1.229** |
| *Age* | 0.021 (0.031) | 0.493 | -0.040 | 0.082 | 0.022 (0.030) | 0.475 | -0.038 | 0.081 |
| *Sex* | 0.096 (0.140) | 0.492 | -0.179 | 0.371 | 0.114 (0.142) | 0.424 | -0.165 | 0.392 |
| *Game Beliefs* | -0.078 (0.208) | 0.707 | -0.486 | 0.330 | -0.069 (0.206) | 0.736 | -0.473 | 0.334 |
| *Organ Donor Register* | -0.038(0.149) | 0.797 | -0.330 | 0.253 | -0.074 (0.148) | 0.619 | -0.364 | 0.217 |
| *Constant* | -0.072 (0.693) | 0.917 | -1.431 | 1.287 | -0.096 (0.687) | 0.889 | -1.443 | 1.251 |
| *McKelvey and Zavoina R^2^* | .015 |  |  |  | .016 |  |  |  |
| *N of Observations (Groups)* | 4,686 (71) |  |  |  | 4,473 (71) |  |  |  |

**Table S6:** *Overall (Model 1) and* *Conditional Cooperation (Model 2) Effects (Random Effects Logit Models with group level clustered random error) on group averages of decisions to register plus Covariates.* The dependent variable is the individual *Decision to Register* (1 = on the register: actively registered under opt-in and not opted-out under opt-out, or 0 = not on the register: not having registered under opt-in and opted-out under opt-out). We have the following independent variables. *Round* is the game number 1-22. *Feedback*: 0 = without individualistic feedback and 1 = with individualistic feedback, *Opt-In:* 0 = Opt-out and = 1 = Opt-in. *Lag Others’ Decisions =* percent of group registered in the previous round. *Sex:* Female = 0, Male = 1. *Game Beliefs:* Not believe it is a game about organ donation = 0, Believe it is a game about organ donation = 1, *Organ Donor Register:* 0 if report not currently registered and 1 if currently registered.

|  | ***Without Individualistic Feedback*** | | | | ***With Individualistic Feedback*** | | | |
| --- | --- | --- | --- | --- | --- | --- | --- | --- |
|  |  |  | ***95%*** |  |  |  | ***95%*** |  |
|  | ***Coefficient (SE_robust_)*** | ***p*** | ***Lower*** | ***Upper*** | ***Coefficient (SE_robust_)*** | ***p*** | ***Lower*** | ***Upper*** |
| *Final Decision to Register* | Model 3 |  |  |  | Model 4 |  |  |  |
| *Round* | **-0.026 (0.012)** | **0.025** | **-0.049** | **-0.003** | -0.022 (0.016) | 0.173 | -0.053 | 0.010 |
| *Opt-In (out)* | -0.009 (0.191) | 0.961 | -0.384 | 0.365 | 0.394 (0.221) | 0.074 | -0.038 | 0.826 |
| *Lag Others’ Decisions* | 0.247 (0.164) | 0.132 | -0.075 | 0.570 | -0.239 (0.133) | 0.074 | -0.500 | 0.023 |
| *Lag Others’ Decisions * Opt-In* | -0.256 (0.151) | 0.091 | -0.552 | 0.041 | 0.172 (0.151) | 0.255 | -0.124 | 0.468 |
| *Age* | 0.021 (0.048) | 0.656 | -0.072 | 0.114 | -0.005 (0.032) | 0.881 | -0.068 | 0.059 |
| *Sex* | 0.188 (0.225) | 0.403 | -0.253 | 0.629 | 0.039 (0.194) | 0.842 | -0.341 | 0.419 |
| *Game Beliefs* | -0.026 (0.318) | 0.934 | -0.650 | 0.597 | -0.241 (0.228) | 0.291 | -0.629 | 0.206 |
| *Organ Donor Register* | -0.241 (0.269) | 0.370 | -0.768 | 0.286 | 0.135 (0.213) | 0.526 | -0.683 | 0.253 |
| *Constant* | -0.360 (1.047) | 0.731 | -2.412 | 1.692 | 0.327 (0.775) | 0.674 | -1.193 | 1.846 |
| *McKelvey and Zavoina R^2^* | .016 |  |  |  | .020 |  |  |  |
| *N of Observations(Groups)* | 2,268 (36) |  |  |  | 2,205 (35) |  |  |  |

**Table S7:** *Across Round Conditional Cooperation Effects as a function of Default and Feedback (Random Effects Logit Models with group level clustered random error) on group averages of decisions to register plus Covariates - the across round Lone-Wolf Effect.* The dependent variable is the individual *Decision to Register* (1 = on the register: actively registered under opt-in and not opted-out under opt-out, or 0 = not on the register: not having registered under opt-in and opted-out under opt-out). We have the following independent variables. *Round* is the game number 1-22, *Opt-In:* 0 = Opt-out and = 1 = Opt-in. *Lag Others’ Decisions =* number of others registered in the previous round. *Sex:* Female = 0, Male = 1. *Game Beliefs:* Not believe it is a game about organ donation = 0, Believe it is a game about organ donation = 1, *Organ Donor Register:* 0 if report not currently registered and 1 if currently registered.

|  | ***With Individualistic Feedback*** | | | |
| --- | --- | --- | --- | --- |
|  |  |  | ***95%*** |  |
|  | ***Coefficient (SE_robust_)*** | ***p*** | ***Lower*** | ***Upper*** |
| *Final Decision to Register* | Model 5 |  |  |  |
| *Round* | -0.010 (0.010) | 0.317 | -0.029 | 0.009 |
| *Opt-In* | **0.860 (0.213)** | **0.000** | **0.442** | **1.278** |
| *Others’ Decisions* | **1.336 (0.183)** | **0.000** | **0.977** | **1.696** |
| *Others’ Decisions * Opt-In* | **-0.543 (0.181)** | **0.003** | **-0.898** | **-0.187** |
| *Age* | -0.009 (0.046) | 0.846 | -0.099 | 0.081 |
| *Sex* | 0.021 (0.260) | 0.937 | -0.488 | 0.530 |
| *Game Beliefs* | -0.319 (0.309) | 0.303 | -0.925 | 0.288 |
| *Organ Donor Register* | 0.218 (0.284) | 0.443 | -0.339 | 0.776 |
| *Constant* | -1.061 (1.019) | 0.298 | -3.058 | 0.936 |
| *McKelvey and Zavoina R^2^* | .185 |  |  |  |
| *N of Observations(Groups)* | 2,310 (35) |  |  |  |

**Table S8:** *Conditional Cooperation Within Rounds (Random Effects Logit Models with group level clustered random error) on group averages of decisions to register plus Covariates - the within round Lone-Wolf Effect.* The dependent variable is the individual *Decision to Register* (1 = on the register: actively registered under opt-in and not opted-out under opt-out, or 0 = not on the register: not having registered under opt-in and opted-out under opt-out). We have the following independent variables. *Round* is the game number 1-22, *Opt-In:* 0 = Opt-out and = 1 = Opt-in, *Others’ Decisions* = number of others registered in the present round. *Sex:* Female = 0, Male = 1. *Game Beliefs:* Not believe it is a game about organ donation = 0, Believe it is a game about organ donation = 1, *Organ Donor Register:* 0 if report not currently registered and 1 if currently registered.

**Modelling Individual Responses (Models 1 to 5 without covariates)**

|  |  |  | ***95%*** |  |  |  | ***95%*** |  |
| --- | --- | --- | --- | --- | --- | --- | --- | --- |
|  | ***Coefficient (SE_robust_)*** | ***p*** | ***Lower*** | ***Upper*** | ***Coefficient (SE_robust_)*** | ***p*** | ***Lower*** | ***Upper*** |
| *Final Decision to Register* | Model 1 |  |  |  | Model 2 |  |  |  |
| *Round* | **-0.026 (0.009)** | **0.006** | **-0.045** | **-0.008** | **-0.024 (0.010)** | **0.012** | **-0.043** | **-0.005** |
| *Feedback* | -0.434 (0.382) | 0.256 | -1.183 | 0.315 | -0.435 (0.396) | 0.272 | -1.211 | 0.340 |
| *Opt-In* | **-0.289 (0.124)** | **0.020** | **-0.531** | **-0.046** | **-0.273 (0.115)** | **0.018** | **-0.499** | **-0.047** |
| *Lag Others’ Decisions* |  |  |  |  | 0.046 (0.087) | 0.594 | -0.217 | 0.124 |
| *Feedback * Opt-In* | **0.782 (0.212)** | **0.000** | **0.366** | **1.198** | **0.827 (0.206)** | **0.000** | **0.423** | **1.230** |
| *Constant* | 0.355 (0.276) | 0.198 | -0.186 | 0.896 | 0.348 (0.288) | 0.228 | -0.217 | 0.913 |
| *McKelvey and Zavoina R^2^* | .014 |  |  |  | .014 |  |  |  |
| *N of Observations (Groups)* | 4,686 (71) |  |  |  | 4,473 (71) |  |  |  |

**Table S9**. *Overall (Model 1) and* *Conditional Cooperation (Model 2) Effects (Random Effects Logit Models with group level clustered random error) on group averages of decisions to register plus Covariates.* The dependent variable is the individual *Decision to Register* (1 = on the register: actively registered under opt-in and not opted-out under opt-out, or 0 = not on the register: not having registered under opt-in and opted-out under opt-out). We have the following independent variables. *Round* is the game number 1-22. *Feedback*: 0 = without individualistic feedback and 1 = with individualistic feedback, *Opt-In:* 0 = Opt-out and = 1 = Opt-in. *Lag Others’ Decisions =* number of others registered in the previous round.

|  | ***Without Individualistic Feedback*** | | | | ***With Individualistic Feedback*** | | | |
| --- | --- | --- | --- | --- | --- | --- | --- | --- |
|  |  |  | ***95%*** |  |  |  | ***95%*** |  |
|  | ***Coefficient (SE_robust_)*** | ***p*** | ***Lower*** | ***Upper*** | ***Coefficient (SE_robust_)*** | ***p*** | ***Lower*** | ***Upper*** |
| *Final Decision to Register* | Model 3 |  |  |  | Model 4 |  |  |  |
| *Round* | -0.026 (0.012) | 0.025 | -0.049 | -0.003 | -0.022 (0.016) | 0.173 | -0.053 | 0.010 |
| *Opt-In* | -0.006 (0.191) | 0.974 | -0.380 | 0.368 | 0.393 (0.220) | 0.074 | -0.038 | 0.825 |
| *Lag Others’ Decisions* | 0.237 (0.164) | 0.147 | -0.084 | 0.558 | 0.241(0.133) | 0.071 | -0.502 | 0.020 |
| *Lag Others’ Decisions * Opt-In* | -0.259 (0.151) | 0.086 | -0.556 | 0.037 | 0.172 (0.150) | 0.250 | -0.121 | 0.4656 |
| *Constant* | 0.090 (0.317) | 0.777 | -0.532 | 0.712 | 0.070 (0.306) | 0.820 | -0.530 | 0.669 |
| *McKelvey and Zavoina R^2^* | .012 |  |  |  | .019 |  |  |  |
| *N of Observations(Groups)* | 2,268 (36) |  |  |  | 2,205 (35) |  |  |  |

**Table S10:** *Across Round Conditional Cooperation Effects as a function of Default and Feedback (Random Effects Logit Models with group level clustered random error) on group averages of decisions to register plus Covariates - the across round Lone-Wolf Effect.* The dependent variable is the individual *Decision to Register* (1 = on the register: actively registered under opt-in and not opted-out under opt-out, or 0 = not on the register: not having registered under opt-in and opted-out under opt-out). We have the following independent variables. *Round* is the game number 1-22, *Opt-In:* 0 = Opt-out and = 1 = Opt-in. *Lag Others’ Decisions =* number of others registered in the previous round.

|  | ***With Individualistic Feedback*** | | | |
| --- | --- | --- | --- | --- |
|  |  |  | ***95%*** |  |
|  | ***Coefficient (SE_robust_)*** | ***p*** | ***Lower*** | ***Upper*** |
| *Final Decision to Register* | Model 5 |  |  |  |
| *Round* | -0.010 (0.010) | 0.319 | -0.029 | 0.009 |
| *Opt-In* | **0.861 (0.216)** | **0.000** | **0.437** | **1.284** |
| *Others’ Decisions* | **1.332 (0.185)** | **0.000** | **0.971** | **1.694** |
| *Others’ Decisions * Opt-In* | **-0.544 (0.184)** | **0.003** | **-0.905** | **-0.183** |
| *Constant* | **-1.454 (0.239)** | **0.000** | **-1.923** | **0.985** |
| *McKelvey and Zavoina R^2^* | .182 |  |  |  |
| *N of Observations(Groups)* | 1,350 (35) |  |  |  |

**Table S11.** *Conditional Cooperation Within Rounds (Random Effects Logit Models with group level clustered random error) on group averages of decisions to register plus Covariates - the within round Lone-Wolf Effect.* The dependent variable is the individual *Decision to Register* (1 = on the register: actively registered under opt-in and not opted-out under opt-out, or 0 = not on the register: not having registered under opt-in and opted-out under opt-out). We have the following independent variables. *Round* is the game number 1-22, *Opt-In:* 0 = Opt-out and = 1 = Opt-in, *Others’ Decisions* = number of others registered in the present round.

**References**

Fischbacher U. (2007). z-Tree: Zurich toolbox for ready-made economic experiments.  *Exp Econ* 10: 171-178. Doi: 10.1007/s10683-006-9159-4

Kessler JB, Roth AE. (2012). Organ allocation policy and the decision to donate.  *Am Econ Rev* 102: 2018-2047. Doi. http://dx.doi.org/10.125/aer.102.5.2018

Tan JHW, Breitmoser Y, Bolle F. (2015). Voluntary contributions by consent or dissent. *Games Econ Behav* 92: 106-121. Doi.  10.1016/j.geb.2015.05.007

1. We can use the Euclidean distance between two states in the action space to determine the closeness of states. [↑](#footnote-ref-1)
2. For generality, Kessler and Roth assume a continuum of players with c ~ F(c). [↑](#footnote-ref-2)
